# The intrinsically disordered E-domains regulate the IGF-1 prohormones stability, subcellular localisation and secretion

**DOI:** 10.1038/s41598-018-27233-3

**Published:** 2018-06-11

**Authors:** Giosuè Annibalini, Serena Contarelli, Mauro De Santi, Roberta Saltarelli, Laura Di Patria, Michele Guescini, Anna Villarini, Giorgio Brandi, Vilberto Stocchi, Elena Barbieri

**Affiliations:** 1 Department of Biomolecular Sciences, University of Urbino Carlo Bo, 61029 Urbino, Italy; 2 Research Department, Fondazione IRCCS Istituto Nazionale dei Tumori, 20133 Milan, Italy; 3 IIM, Interuniversity Institute of Myology, 61029 Urbino, Italy

## Abstract

Insulin-like growth factor-1 (IGF-1) is synthesised as a prohormone (proIGF-1) requiring enzymatic activity to yield the mature IGF-1. Three proIGF-1s are encoded by alternatively spliced IGF-1 mRNAs: proIGF-1Ea, proIGF-1Eb and proIGF-1Ec. These proIGF-1s have a common IGF-1 mature sequence but different E-domains. The structure of the E-domains has not been resolved, and their molecular functions are still unclear. Here, we show that E-domains are Intrinsically Disordered Regions that have distinct regulatory functions on proIGF-1s production. In particular, we identified a highly conserved N-glycosylation site in the Ea-domain, which regulated intracellular proIGF-1Ea level preventing its proteasome-mediated degradation. The inhibition of N-glycosylation by tunicamycin or glucose starvation markedly reduced proIGF-1Ea and mature IGF-1 production. Interestingly, 2-deoxyglucose, a glucose and mannose analogue, increased proIGF-1Ea and mature IGF-1 levels, probably leading to an accumulation of an under-glycosylated proIGF-1Ea that was still stable and efficiently secreted. The proIGF-1Eb and proIGF-1Ec were devoid of N-glycosylation sites, and hence their production was unaffected by N-glycosylation inhibitors. Moreover, we demonstrated that alternative Eb- and Ec-domains controlled the subcellular localisation of proIGF-1s, leading to the nuclear accumulation of both proIGF-1Eb and proIGF-1Ec. Our results demonstrated that E-domains are regulatory elements that control IGF-1 production and secretion.

## Introduction

Insulin-like growth factor-1 (IGF-1) is a growth factor with multiple roles in various aspects of normal and pathological growth and differentiation^1,2^. The translation of the IGF-1 gene gives rise to an immature IGF-1 peptide, which has a signal peptide at the 5′ end of the gene, a core region and a C-terminal E-domain extension. The passage of the polypeptide into the endoplasmic reticulum (ER) removes the signal peptide, while the nascent IGF-1 prohormone (proIGF-1) is emerging, retaining the E-domain. Conversion of proIGF-1 to mature peptide requires the endoproteolytic cleavage of the E-domain by proprotein convertases, such as furin, which processes proproteins at highly conserved, unique pentabasic motif^3,4^.

Due to alternative splicing of terminal exon 5 of the IGF-1 gene, three distinct proIGF-1s might exist: proIGF-1Ea, proIGF-1Eb and proIGF-1Ec^3–5^. These prohormones have the same IGF-1 mature sequence of 70 amino acids (aa) but different E-domains. In particular, the human Ea-domain is composed of 35 aa; the first 16 aa of Ea-domain are common in all E-domains, while 19 aa are unique to this isoform. The human Ea-domain contains a potential N-glycosylation site, N92, which follows the consensus sequence motif for N-glycosylation, NX(S/T) (where X can be any amino acid except proline). Accordingly, both unglycosylated proIGF-1Ea (11.7 kDa) and glycosylated proIGF-1Ea (~17–22 kDa) were found in normal and IGF-1-overexpressing cells^6,7^. The human Eb- and Ec-domains contain the 16 common aa and 61 and 24 additional isoform-specific aa respectively, with a predicted molecular weight of 16.5 kDa for proIGF-1Eb and 12.5 kDa for proIGF-1Ec. The human Eb- and Ec-domains lack potential N-linked glycosylation consensus sequences^3,4^.

Previously termed “inactive precursors”, proIGF-1s are currently recognised as stable intermediates of posttranslational processing. Accordingly, under physiological condition mammalian tissues mainly produced the glycosylated proIGF-1Ea^6^. Moreover, several studies demonstrated that proIGF-1s remained unprocessed in cultured cells, whether endogenously expressed IGF-1 isoforms (HepG2, K562 and HeLa cells^8,9^) or that were exogenously transfected with IGF-1 isoforms (HEK293 cells^7^). More interestingly, a differential expression pattern of the proIGF-1s was reported in normal *versus* cancer tissues^8,10,11^.

Whether the alternative E-domains might regulate proIGF-1s is still an open question^4^. In mouse skeletal muscle, viral delivery of IGF-1Ea and IGF-1Ec, but not mature IGF-1, increases muscle mass. Hence, the E-domains are necessary to promote the local production of IGF-1 and functional hypertrophy^12^.

We and others have recently shown that the distribution of intrinsic disorder propensity within the amino acid sequence of mature IGF-1 is markedly different compared to E-domains^5,13^. In particular, bioinformatic analysis of proIGF-1 structures showed that the E-domains were putative intrinsically disordered regions (IDRs). IDRs are regions within proteins that exhibit high flexibility and may lack a secondary or tertiary structure^14^. It is worth mentioning that also the other two members of the IGF family, proinsulin and proIGF-2, possess IDRs, although the degree of disorder across the IGF family varies significantly^13^. Despite the fact that amino acid sequences of E-domains are less conserved than those of mature IGF-1, we demonstrated that the disordered propensity of E-domains has been strongly conserved^5^. In fact, IDRs can tolerate a higher number of mutations without substantial loss of flexibility and function^15,16^.

IDRs may facilitate the regulation of protein function through various mechanisms. For example, owing to their conformational flexibility, IDRs have a high propensity to undergo posttranslational modifications, such as acetylation, glycosylation, methylation, or phosphorylation^15,17^. IDRs might also control protein half-life by efficiently engaging proteins to the proteasome^18,19^. Moreover, studies have identified IDRs as enriched in the alternatively spliced protein segments, indicating that protein isoforms may display functional diversity due to the alteration of tissue-specific and species-specific modules within these regions^20^.

In this study, we analysed the structural proprieties of proIGF-1s, using a combination of bioinformatics analyses and limited proteolysis. Site-direct mutagenesis and inhibition of N-glycosylation were used to evaluate the role of glycosylation on proIGF-1s regulation in terms of stability and secretion. Finally, we investigated the role of alternative E-domains on proIGF-1s subcellular localisation. Our results show that the alternative disordered E-domains affect distinct aspects of proIGF-1s regulation including protein stability, localisation and secretion. Thus, E-domains may represent novel targets to control proIGF-1s and, by extension, mature IGF-1 production.

## Results

### Disorder propensity of Human proIGF-1s

**Figure 1:**
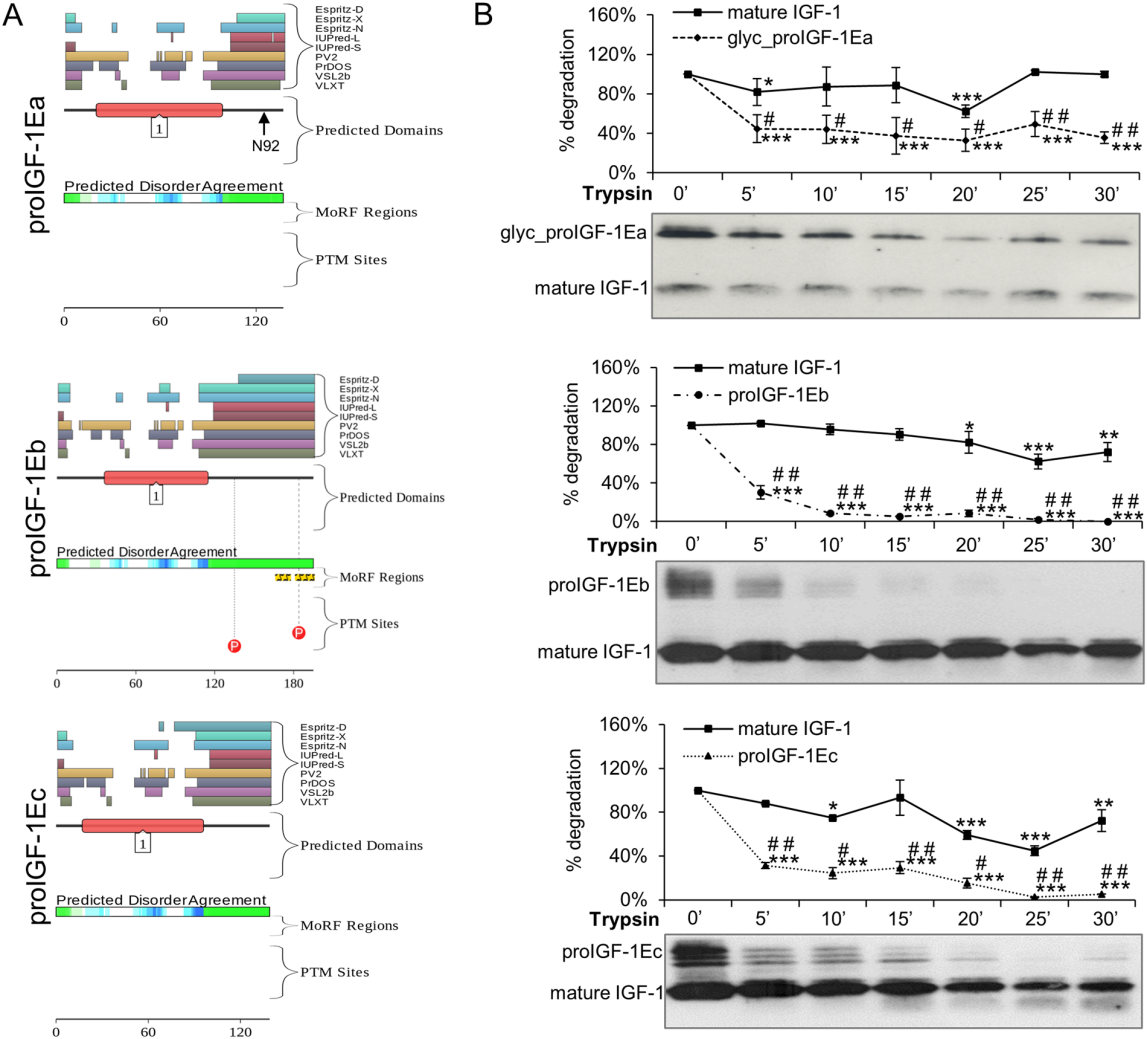
Evaluation of the intrinsic disorder propensity of human proIGF-1s predicted using the D^2^P^2^ platform (**A**) and limited proteolysis (**B**). (**A**) Analysis of the intrinsically disordered regions of human proIGF-1Ea (ENSP00000416811), proIGF-1Eb (ENSP00000302665) and proIGF-1Ec (ENSP00000376638) sequences by the D^2^P^2^ platform (http://d2p2.pro/). The red box corresponds to the insulin-like domain; the green-and-white bar in the middle of the plot shows the predicted disorder agreement among nine predictors, with the green parts corresponding to the portions of sequence where at least 75% of the predictors agreed. Yellow bars show the location of the predicted disorder-based binding sites (molecular recognition features, MoRFs), whereas red circles at the bottom of the plot show the location of putative phosphorylation sites. Position of the N-glycosylation site of Ea-domain (N92) is also indicated. (**B**) Limited proteolysis of proIGF-1s. Cell culture supernatants of IGF-1Ea-, IGF-1Eb- or IGF-1Ec-transfected HEK293 cells were concentrated using Amicon Ultra 3 K centrifugal filters and incubated with 0.2 μM trypsin at 37 °C for different times. Reactions were removed over a time-course and the digested products were loaded on 12% SDS-PAGE and analysed by western blotting with an anti-mature IGF-1 antibody. Results are means ± SEM (n = 3). Repeated measures ANOVA, ^#^(*p* < 0.01) and ^##^(*p* < 0.0001) significantly different compared to mature IGF-1; *(*p* < 0.05), **(*p* < 0.001) and ***(*p* < 0.0001) significantly different compared to the 0-minute time point. Cropped blots are shown. Uncropped blots are presented in Supplementary Fig. S1A.

We used the D^2^P^2^ platform (http://d2p2.pro/) and limited proteolysis to predict intrinsically disordered regions of human proIGF-1Ea (ENSP00000416811), proIGF-1Eb (ENSP00000302665) and proIGF-1Ec (ENSP00000376638)^21,22^ (Fig. 1). Figure 1A shows the plot generated by the D^2^P^2^ platform: this analysis showed that the mature IGF-1 is mostly ordered, while all E-domains were predicted to contain disordered residues. Moreover, the Eb-domain also contains two predicted molecular recognition features (MoRFs) and two phosphorylation sites.

Subsequently, we used limited proteolysis to identify the regions of the polypeptide chain mostly prone to proteolysis and thus the sites of high flexibility or local unfolding^22^ (Fig. 1B). The supernatant of HEK293 cells enriched with glycosylated proIGF-1Ea, proIGF-1Eb, proIGF-1Ec and mature IGF-1 were digested with trypsin, loaded on SDS-PAGE gels and probed with the anti-mature IGF-1 antibody. As shown in Fig. 1B, all proIGF-1s were sensitive to trypsin digestion while mature IGF-1 was significantly more resistant. Digestion of mature IGF-1 required long term incubation with trypsin (i.e. >45 minutes) (Supplementary Fig. S1B). Similar results were obtained using proteinase K digestion of proIGF-1Ea (Supplementary Fig. S1C). These data show that proIGF-1s are composed of both protein structural domain, i.e. the mature IGF-1, and intrinsically disordered regions, i.e. the C-terminal E-domains.

### Intracellular IGF-1 is mainly expressed as proIGF-1Ea, not mature IGF-1

**Figure 2:**
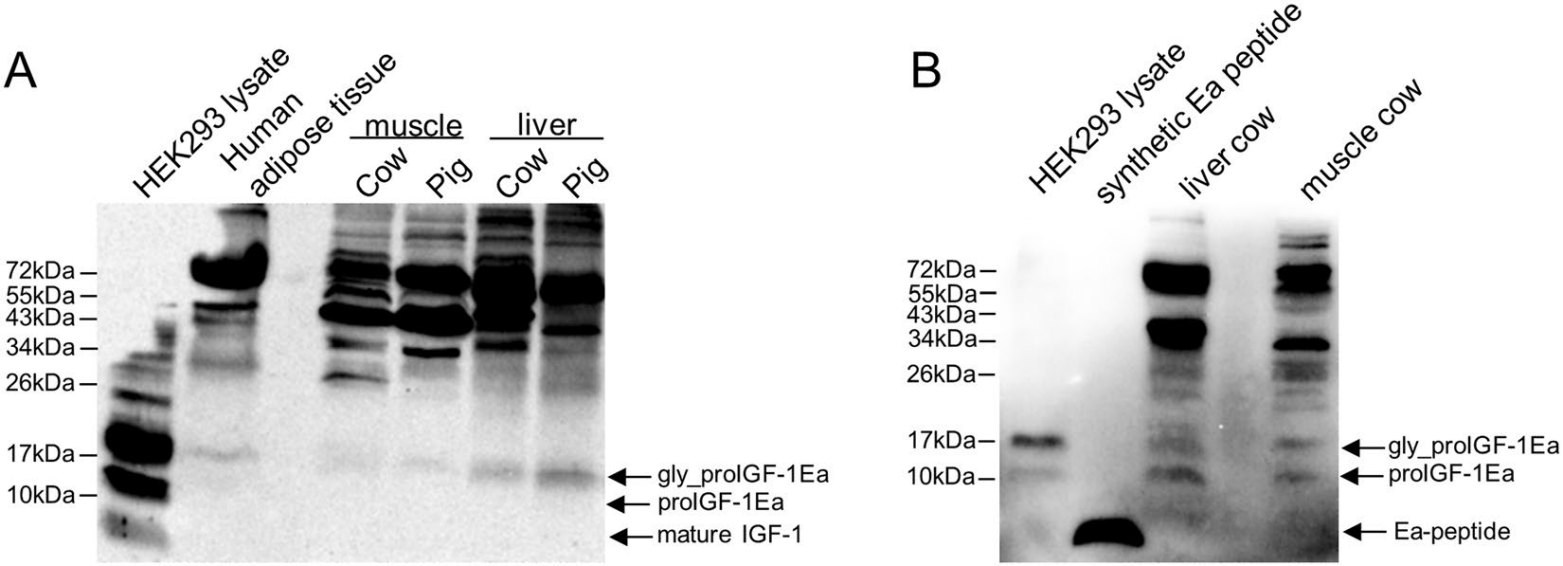
Immunoblotting of several mammalian tissues using an antibody directed against mature IGF-1 sequence (**A**) or common region of E-peptides (**B**). (**A**) Protein lysates (80 μg) were subjected to 12% SDS-PAGE and immunoblotted with anti-mature IGF-1 antibody. A band at a molecular weight around 17 kDa, most likely representing glycosylated proIGF-1Ea, was detected in all the tissue samples tested. A cell lysate of HEK293 overexpressing IGF-1Ea was used has a positive control. The band corresponding to mature IGF-1 (~7 kDa) was not found in tissues and was detectable in HEK293 overexpressing IGF-1Ea only after long exposure of the blots. (**B**) Immunoblotting of liver and muscle cow lysate using an antibody directed against E-domains. Two bands at a molecular weight around 12 kDa and 17 kDa were detected in all the tissue samples tested, most likely representing the unglycosylated and glycosylated proIGF-1Ea respectively. No band at a molecular weight of Ea peptide (~4 kDa) was detected. A cell lysate of HEK293 overexpressing IGF-1Ea and synthetic human Ea peptide were used as positive controls.

Using RT-PCR analyses, we previously demonstrated that skeletal muscles, adipose tissues and liver of several mammalian species mainly expressed the IGF-1Ea isoform, which represents about 90% of IGF-1 transcripts^5^. The first goal of the present study was to examine the protein forms endogenously produced in these tissues. Immunoblotting of protein lysates using the anti-mature IGF-1 antibody showed a distinct ~17 kDa band, most likely representing glycosylated proIGF-1Ea, in all samples analysed (Fig. 2A). Notably, the band corresponding to mature IGF-1 (~7 kDa) was not found in naïve tissues, in agreement with Durzynska, J. *et al*.^6^.

To further confirm the presence of proIGF-1s, we used specific antibody directed against the common E-domain region of proIGF-1s (RSVRAQRHTD). The antibody specificity towards E-domain region was checked using HEK293 cells overexpressing IGF-1 isoforms (Supplementary Fig. S2). As shown in Fig. 2B, two bands of ~12 kDa and ~17 kDa were detected with the anti E-domain antibody, corresponding respectively to the molecular weight of unglycosylated and glycosylated proIGF-1Ea. As expected, no band corresponding to the molecular size of Ea-domain (~4 kDa) was detected in the lysate of HEK293 cells overexpressing IGF-1Ea or tissues confirming that the E-domain was not cleaved intracellularly from the IGF-1 mature protein. Subsequently, we moved to a cell-based system to improve IGF-1 detection and to control the IGF-1 isoforms produced. We recently demonstrated that in HEK293 cells over-expressing IGF-1 isoforms the proIGF-1s are the main forms produced intracellularly, and for proIGF-1Ea both unglycosylated (~12 kDa) and glycosylated (~17 kDa) forms were detected^7^. Hence the tissue expression pattern of IGF-1 was recapitulated in our HEK293 cell-based transient gene expression system.

We next examined the effects of E-domains on proIGF-1s stability and secretion, starting with the predominant isoform produced in normal tissues, i.e. the proIGF-1Ea.

### Glycosylation is necessary to stabilise proIGF-1Ea and regulate mature IGF-1 secretion

**Figure 3:**
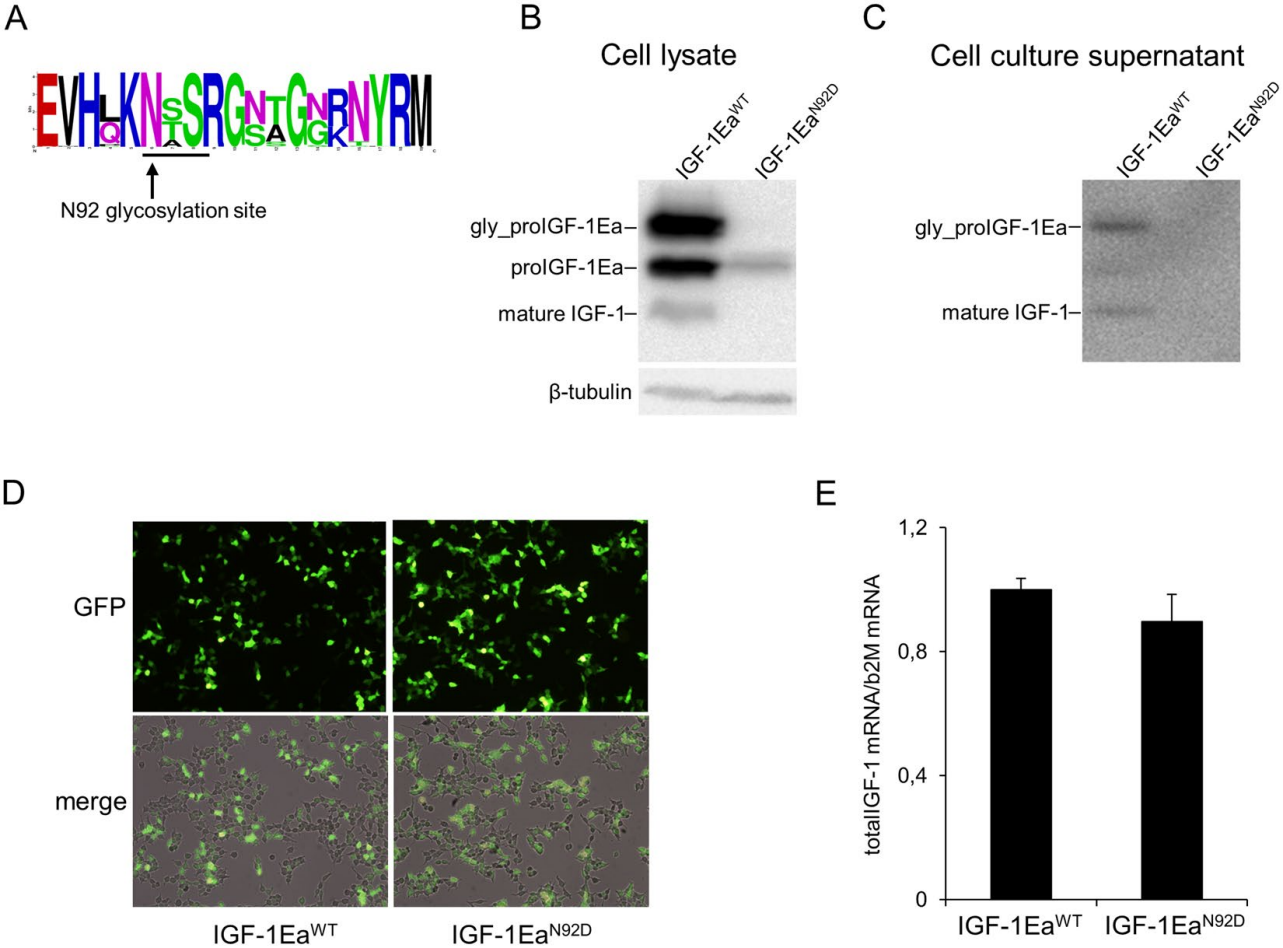
Conservation of the N-glycosylation site of Ea-domain (N92) (**A**) and effect of site-direct mutation of this N-glycosylation site (IGF-1Ea^N92D^ mutant) on intracellular (**B**) or extracellular (**C**) proIGF-1Ea production. Comparison of transfection efficiency between wild-type (IGF-1Ea^WT^) and IGF-1Ea^N92D^ constructs (**D** and **E**). (**A**) WebLogo of Ea-domain sequences obtained from UniProt database. The relative frequency plots of amino acids of 250 E-domain sequences obtained from UniProt database is shown. The consensus sequence motif for N-glycosylation NX(S/T) (where X can be any amino acid except proline) is underline. The WebLogo was produced using the web server at http://weblogo.berkeley.edu/logo.cgi. (**B** and **C**) Transfection of HEK293 cells with IGF-1Ea^WT^ or IGF-1Ea^N92D^. IGF-1Ea^WT^ and IGF-1Ea^N92D^ were transiently expressed in HEK293 cells. After 24 h the cell lysates (**B**) and cell culture supernatants (**C**) were analysed by western blot using an antibody directed against mature IGF-1 sequence. There was no significant difference in GFP fluorescence intensity (10 × magnification) (**D**) and total IGF-1 mRNA quantity (*p* = 0.314) (**E**) between the IGF-1Ea^WT^ and IGF-1Ea^N92D^ constructs. Cropped blots are shown. Uncropped blots are presented in Supplementary Fig. S3.

Multiple sequence alignment of vertebrate Ea-domain showed that the N-glycosylation site of proIGF-1Ea has been conserved from teleosts to mammals (Fig. 3A). This strong evolutionary conservation led us to hypothesise that glycosylation could play an important role in the regulation of proIGF-1Ea.

**Figure 4:**
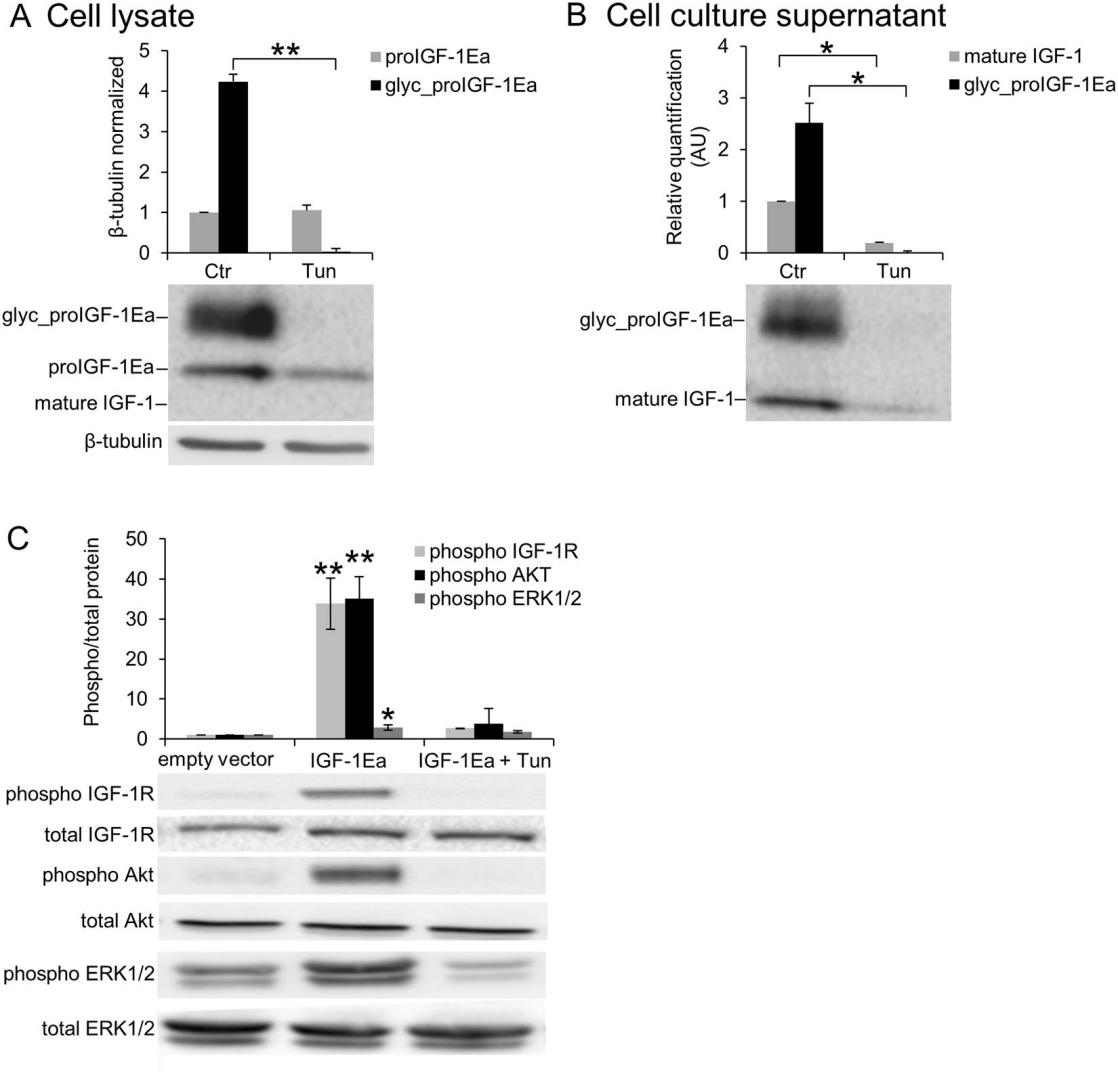
Effect of tunicamycin (Tun) treatment on proIGF-1Ea glycosylation. (**A** and **B**) IGF-1Ea was transiently expressed in HEK293 cells in the presence of 0.1 µg/ml of Tun. After 24 h the cell lysates (**A**) and cell culture supernatants (**B**) were analysed by western blot and relative expression level of glycosylatyed proIGF-1Ea, unglycosylated proIGF-1Ea and mature IGF-1 was calculated. The band at a molecular weight of ~17 kDa, corresponding to glycosylated proIGF-1Ea, disappeared in presence of Tun in cell lysates (**A**) and in cell culture supernatants (**B**). The band corresponding to mature IGF-1 (~7 kDa) was markedly reduced in cell culture supernatants after Tun treatment (**B**). (**C**) Phosphorylation of IGF-1R, AKT and ERK1/2 after treatment of MCF-7 cells with cell culture supernatants from IGF-1Ea-transfected HEK293 cells treated with Tun. The phosphorylation of the IGF-1R pathway was markedly reduced by Tun treatment. β-tubulin was used as a loading control for the cell lysates. Results are means ± SEM (n = 3); T-test or a one-way ANOVA was used to evaluate statistical significance (*p < 0.01, **p < 0.0001). Cropped blots are shown. Uncropped blots are presented in Supplementary Fig. S4.

Site-direct mutation of this glycosylation site (IGF-1Ea^N92D^) resulted in a ~12 kDa band, corresponding to the size of unglycosylated proIGF-1Ea (Fig. 3B). Western blotting analysis showed that the intracellular level of unglycosylated proIGF-1Ea was significantly lower in IGF-1Ea^N92D^-transfected HEK293 cells compared to wild-type IGF-1Ea (IGF-1Ea^WT^) (Fig. 3B). Moreover, contrary to IGF-1Ea^WT^, the IGF-1Ea^N92D^-transfected HEK293 cells did not secrete IGF-1 (both glycosylated proIGF-1Ea and mature IGF-1) (Fig. 3C). Notably, similar GFP fluorescence intensity (Fig. 3D) and total IGF-1 mRNA (Fig. 3E; *p* = 0.314) were found in IGF-1Ea^WT^- and IGF-1Ea^N92D^-transfected HEK293 cells, ruling out the possibility that the marked reduction in the protein levels between the two constructs was due to different transfection efficiency. Thus, these data suggest that glycosylation of proIGF-1Ea is required for efficient IGF-1 production and secretion. Subsequently, we wondered whether direct inhibition of N-glycosylation by tunicamycin (Tun) might interfere with IGF-1 production. Notably, the band corresponding to glycosylated proIGF-1Ea disappeared after treatment with Tun (Fig. 4A). Moreover, the analysis of cell culture supernatants of the IGF-1Ea-transfected HEK293 cells showed that Tun treatment completely abrogated the glycosylated proIGF-1Ea secretion and markedly reduced the secretion of mature IGF-1 (Fig. 4B). The marked reduction of glycosylated proIGF-1Ea after Tun treatment was not due to general suppression of transcription, as shown by total IGF-1 mRNA quantification (Supplementary Fig. 4C), or general protein synthesis inhibition, as shown by co-transfection of GFP (Supplementary Fig. 4D). Notably, conditioned media from IGF-1Ea-transfected HEK293 cells treated with Tun was unable to activate the IGF-1 receptor (IGF-1R) and downstream phosphorylation of ERK1/2 and AKT of MCF-7 breast cancer cells (Fig. 4C).

Similar results were obtained by blocking N-glycosylation by glucose withdrawal although the effect was less specific since glucose starvation also slightly decreased cell number and GFP-dependent fluorescence (Supplementary Fig. 5).

Collectively, these results documented that the interference with Ea-domain glycosylation resulted in a dramatic decrease of intracellular proIGF-1Ea level and hence proIGF-1Ea and mature IGF-1 secretion.

### The turnover of unglycosylated proIGF-1Ea is faster than glycosylated proIGF-1Ea and depends on proteasome activity

**Figure 5:**
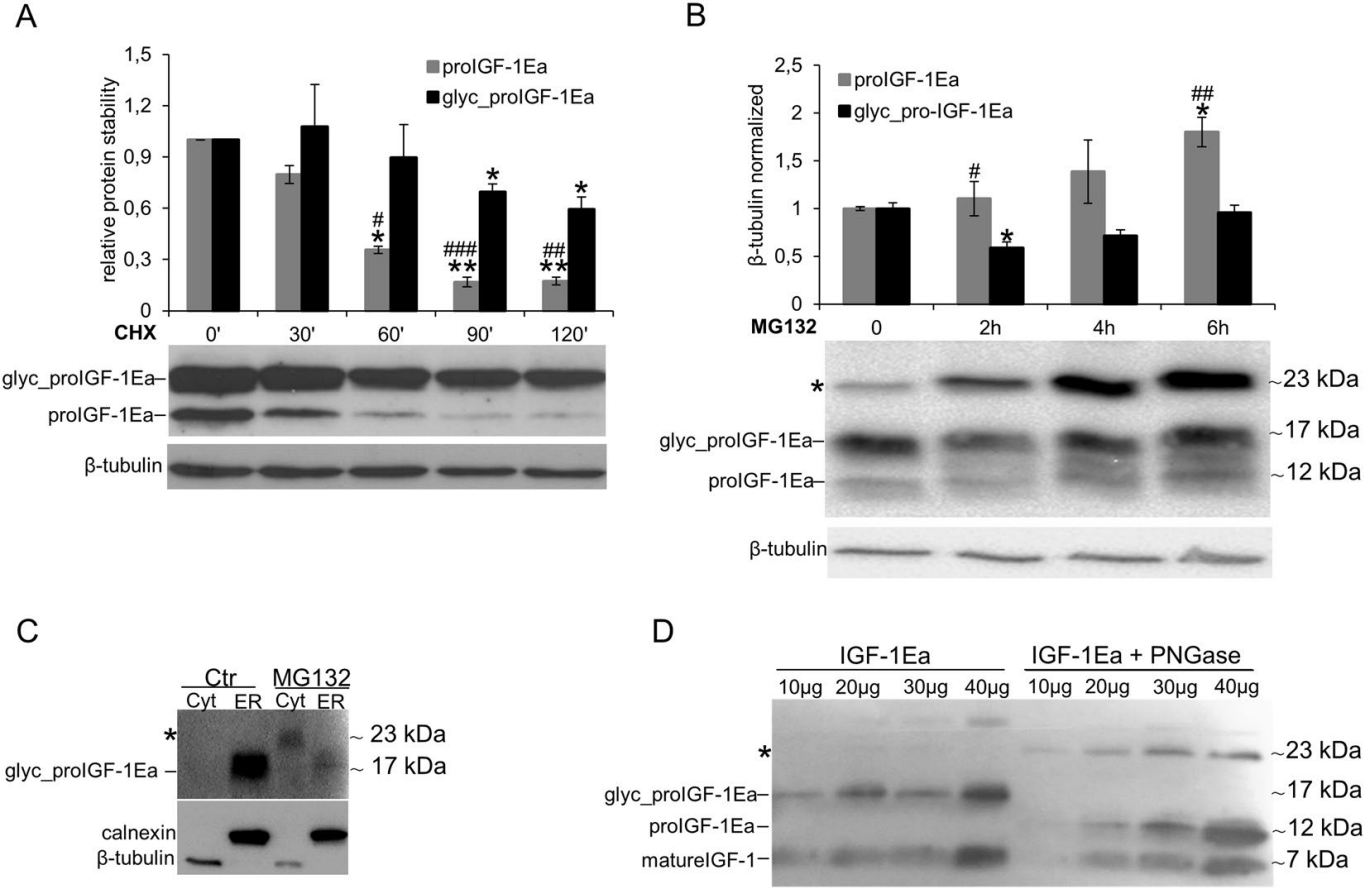
Analysis of unglycosylated and glycosylated proIGF-1Ea turnover. (**A** and **B**) IGF-1Ea was transiently expressed in HEK293 cells in the presence of 25 μg/ml of protein synthesis inhibitor cycloheximide (CHX) (**A**) or 10 μM of the proteasome inhibitor MG132 (**B**) in a time-course experiment. Cells were collected at different time points, and relative expression level of glycosylated and unglycosylated proIGF-1Ea was calculated. After MG132 treatment intracellular accumulation of a ~23 kDa band was found (indicated with an asterisk in **B**), probably representing unglycosylated proIGF-1Ea dimer. (**C**) Cytosol (Cyt) and endoplasmic reticulum (ER) isolations of IGF-1Ea-transfected HEK293 cells treated with MG132. The band at a molecular weight around 23 kDa was detected only after MG132 treatment and only in the cytosolic fraction. (**D**) Deglycosylation of proIGF-1Ea enriched media using the *N*-Glycosidase F (PNGase F). The PNGase treatment determined an accumulation of the band at a molecular weight around 23 kDa probably corresponding to unglycosylated pro-IGF-1Ea dimer. Results are means ± SEM (n = 3). Repeated measures ANOVA, ^#^(p < 0.05), ^##^(p < 0.01) and ^###^(p < 0.0001) significantly different compared to glycosylated proIGF-1Ea; *(p < 0.01) and **(p < 0.0001) significantly different compared to the 0-minute time point. β-tubulin was used as a loading control for the cell lysates and the cytosol separation; calnexin was used as a control for the ER separation. Cropped blots are shown. Uncropped blots are presented in Supplementary Fig. S6.

The inhibition of proIGF-1Ea glycosylation by mutation of the glycosylation site (proIGF-1Ea^N92D^) (Fig. 3B), Tun treatment (Fig. 4A) or glucose starvation (Supplementary Fig. 5A), did not determine a concomitant increase of unglycosylated proIGF-1Ea. One possibility is that the unglycosylated proIGF-1Ea is rapidly degraded. Thus, we next sought to examine the role of glycosylation in the stability of proIGF-1Ea. In presence of cycloheximide (CHX), a protein synthesis inhibitor, the turnover rate of unglycosylated proIGF-1Ea was faster than glycosylated one (Fig. 5A). To verify the involvement of 26 S proteasome machinery on unglycosylated proIGF-1Ea degradation, we subsequently treated IGF-1Ea-transfected HEK293 cells with proteasome inhibitor MG132. As shown in Fig. 5B, we found an increase of unglycosylated proIGF-1Ea, while glycosylated proIGF-1Ea was only marginally affected by the proteasome inhibitor. These results demonstrated that unglycosylated proIGF-1Ea was unstable and degraded faster than glycosylated proIGF-1Ea.

Besides the increase of unglycosylated proIGF-1Ea, treatment with MG132 also promoted an accumulation of a band of ~23 kDa (indicated with an asterisk in Fig. 5B). This band was approximately twice the molecular weight of the unglycosylated proIGF-1Ea monomer (11.7 kDa) and was detected with both the anti-mature IGF-1 antibody (Fig. 5B) and the anti-E-domain antibody (Supplementary Fig. S6E). Thus, we hypothesised that inhibition of the proteasome leads to unglycosylated proIGF-1Ea accumulation and dimerisation. In support of this hypothesis, we found that proteasome inhibition by MG132 increased the cytoplasmic level of the ~23 kDa protein (Fig. 5C). Moreover, PNGase deglycosylation of the supernatant of HEK293 cells enriched with glycosylated proIGF-1Ea increased both the unglycosylated proIGF-1Ea and the putative ~23 kDa dimer in the cell culture supernatants (Fig. 5D). Taken together, these data showed that addition of N-glycan on N92 site of Ea-domain prevented the degradation of proIGF-1Ea by the proteasome, probably overcoming the folding limitation of Ea-domain and the tendency of unglycosylated proIGF-1Ea to self-aggregate.

### The glucose analogue, 2-deoxyglucose, rescues the secretion defect of IGF-1 under low glucose conditions

**Figure 6:**
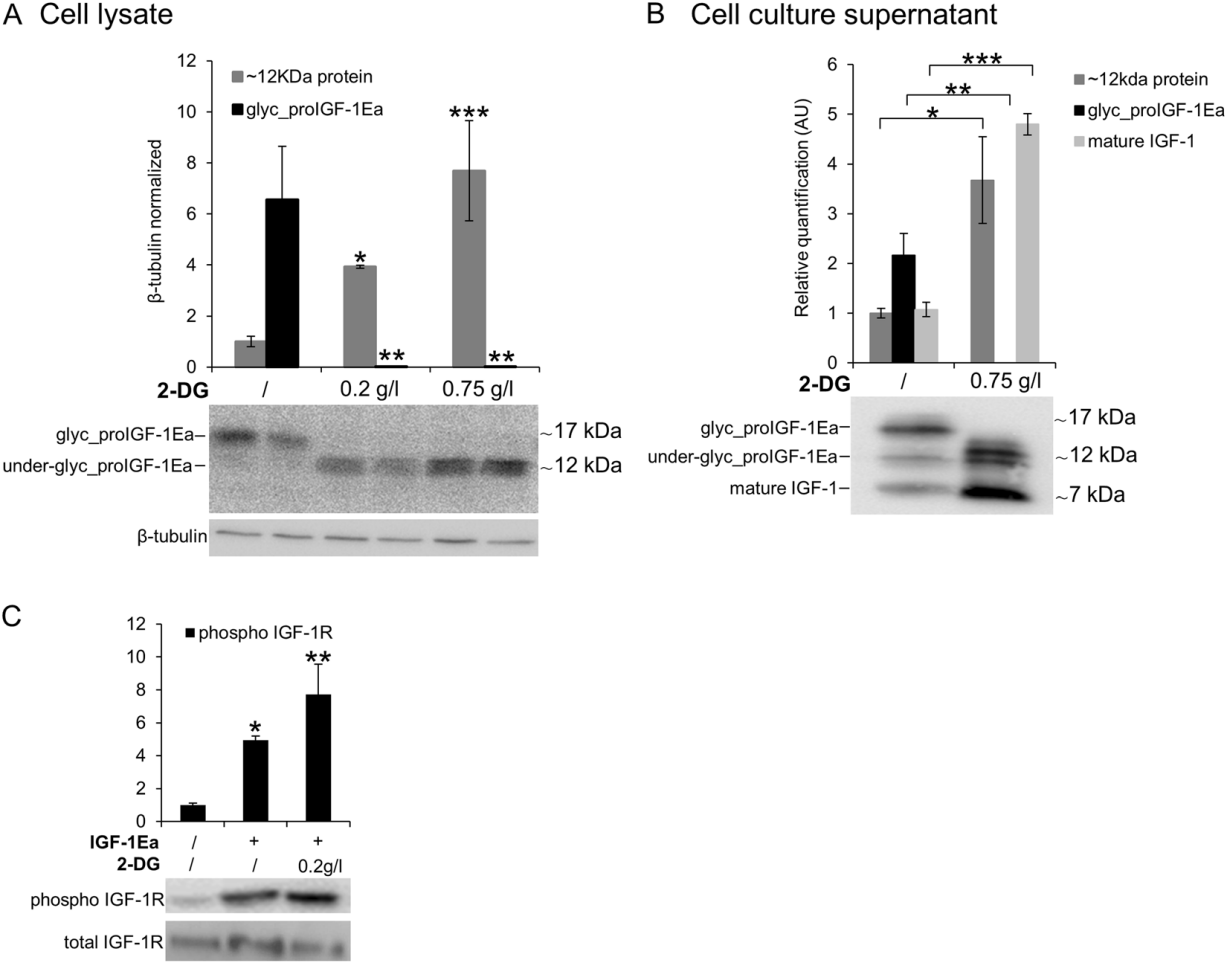
Effect of 2-Deoxyglucose (2-DG) on glycosylated proIGF-1Ea production. (**A** and **B**) IGF-1Ea was transiently expressed in HEK293 cells growth in low glucose medium (0.65 g/L) with or without 2-DG. After 24 h the cell lysates (**A**) and cell culture supernatants (**B**) were analysed by western blot and relative expression level of glycosylated proIGF-1Ea, unglycosylated proIGF-1Ea and mature IGF-1 was calculated. The band at a molecular weight around 17 kDa, corresponding to glycosylated proIGF-1Ea, disappeared in the presence of 2-DG in the cell lysates (**A**) and in the cell culture supernatants (**B**). 2-DG treatment determined also an intracellular (**A**) and supernatants (**B**) accumulation of a ~12 kDa band, probably representing an under-glycosylated proIGF-1Ea form. (**C**) Phosphorylation of IGF-1R after treatment of MCF-7 cells with conditioned media from IGF-1Ea-transfected HEK293 cells treated with 2-DG. Phosphorylation of IGF-1R increased both in 2-DG-treated and untreated cells. β-tubulin was used as a loading control for the cell lysates. Results are means ± SEM (n = 3); T-test or a one-way ANOVA was used to evaluate statistical significance (*p < 0.05, **p < 0.01 and ***p < 0.0001). Cropped blots are shown. Uncropped blots are presented in Supplementary Fig. S7.

Due to its inhibitory activity on glycolysis, 2-deoxyglucose (2-DG) has been shown to selectively kill cancer cells^23,24^. Because of its similarity to mannose, 2-DG is also known to interfere with N-linked glycosylation^24–26^. Notably, increasing evidence links 2-DG effects to the interference with glycosylation instead of glycolysis inhibition^27–29^. Prompted by this, we investigated whether 2-DG interfered with proIGF-1Ea glycosylation. IGF-1Ea-transfected HEK293 cells were grown in low glucose medium (0.65 g/L) in presence of increasing concentration of 2-DG (Fig. 6). As shown in Fig. 6A, the band of glycosylated proIGF-1Ea (~17 kDa) disappeared after the addition of 0.2 g/L or 0.75 g/L of 2-DG. Notably, we also found a dose-dependent accumulation of a ~12 kDa band, both intracellularly (Fig. 6A) and extracellularly (Fig. 6B). Both the anti-mature IGF-1 antibody (Fig. 6A) and the anti-E-domain antibody (Supplementary Fig. 7D) recognised this ~12 kDa band. Thus, it is likely that the 12kD protein was not a non-specific product that appears after 2-DG treatment. Moreover, cell culture supernatant from IGF-1Ea-transfected HEK293 cells treated with 2-DG fully activated the IGF-1R of MCF-7 breast cancer cells (Fig. 6C). Thus, these data indicate that 2-DG interfered with the proIGF-1Ea glycosylation leading to an accumulation of a ~12 kDa protein, probably representing an aberrant under-glycosylated form of proIGF-1Ea, which was stable and efficiently secreted. These results are in line with a recent study showing that the effect of 2-DG on protein glycosylation was dose-dependent and, at doses similar to those used in the present study, 2-DG increased mannose incorporation into cellular glycoproteins instead of inhibits glycosylation^26^. Further studies are needed to identify the glycan structure of proIGF-1Ea that appears after 2-DG treatment and to understand how 2-DG rescues the secretion defect of proIGF-1Ea under low glucose conditions.

### The presence of the alternative Eb- and Ec-domains hampers the proIGF-1Eb and proIGF-1Ec sensitivity to N-glycosylation inhibitors and determines the nuclear accumulation of prohormones

**Figure 7:**
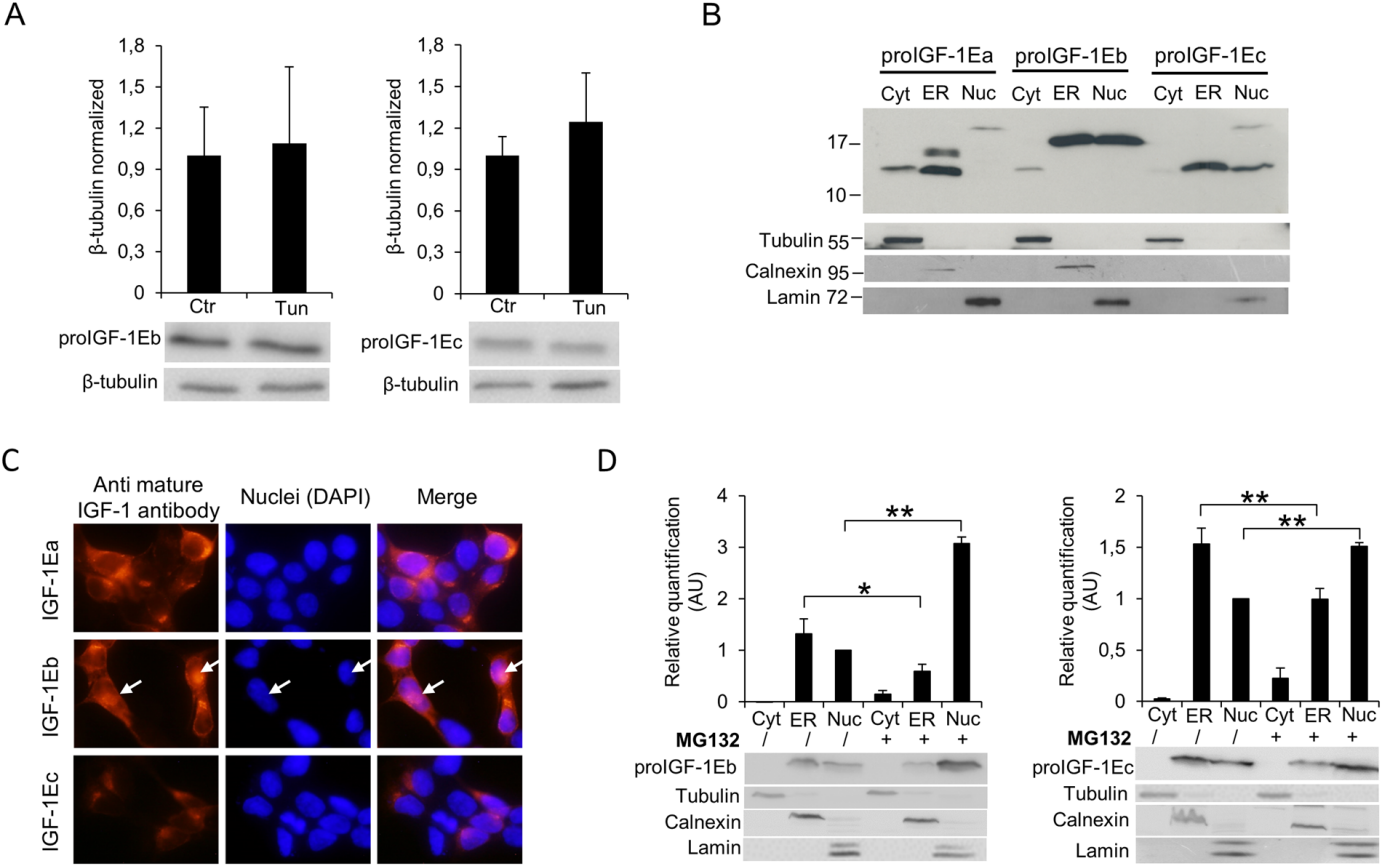
Effect of inhibition of N-glycosylation using Tun on IGF-1Eb (A, left panel) and IGF-1Ec (A, right panel) production and subcellular localisation of proIGF-1s in untreated (**B** and **C**) or MG132 treated HEK293 cells (**D**). (**A**) IGF-1Eb or IGF-1Ec were transiently expressed in HEK293 cells in the presence of 0.1 µg/ml of Tun. After 24 h the cell lysates were analysed by western blot and relative expression level of proIGF-1Eb (left panel) and proIGF-1Ec (right panel) was calculated. The molecular weight and the expression level of proIGF-1Eb (*p* = 0.8) and proIGF-1Ec (*p* = 0.6) were unaffected by Tun treatment. Results are means ± SEM (n = 3); T-test was used to evaluate statistical significance. (**B**) Subcellular localization of IGF-1 isoforms analysed by cytosol, ER and nucleus isolations or (**C**) immunofluorescence staining of IGF-1Ea-, IGF-1Eb- or IGF-1Ec-transfected HEK293 cells. ProIGF-1Ea was mainly localised in the cytosol fraction (unglycosylated proIGF-1Ea) and the ER fraction (both unglycosylated and glycosylated proIGF-1Ea) while proIGF-1Eb and proIGF-1Ec were mainly localised in the nuclear fraction and ER. (**D**) Subcellular localisation of proIGF-E1b (left panel) or proIGF-1Ec (right panel) after treatment with 10 μM of the proteasome inhibitor MG132 for 6 h. Results are means ± SEM (n = 3); a two-way ANOVA was used to evaluate statistical significance (*p < 0.01 and **p < 0.001). β-tubulin was used as a loading control for the cell lysates and the cytosol separation; calnexin as a control for the ER separation and lamin as a control for the nucleus separation. DAPI nuclear stain (blue). Cropped blots are shown. Uncropped blots are presented in Supplementary Fig. S8.

The proIGF-1Eb and proIGF-1Ec ran at the expected molecular weight of ~16.5 kDa and ~12.5 kDa in SDS-PAGE gels respectively, suggesting that these prohormones did not undergo posttranslational modification (Supplementary Fig. S2)^7^. Accordingly, unlikely proIGF-1Ea, proIGF-1Eb and proIGF-1Ec did not contain any potential glycosylation sites and migrated at the same molecular weight after treatment of HEK293 cells with Tun (Fig. 7A) or no glucose media (Supplementary Fig. S8C). Noteworthy, unlikely proIGF-1Ea, also the intracellular levels of proIGF-1Eb and proIGF-1Ec were unaffected by Tun treatment (Fig. 7A). Hence, the presence of the Eb- or Ec-domains, instead of Ea-domain, completely abrogated the response of proIGF-1s to glycosylation inhibitors.

Notably, previous studies demonstrated that both proIGF-1Eb and proIGF-1Ec might localise in the nucleus^30–32^. This led us to hypothesise that disordered Eb- and Ec-domains might control subcellular localisation of proIGF-1Eb and proIGF-1Ec. To test this hypothesis, we investigated the subcellular localisation of proIGF-1s by cytosol, ER and nucleus isolations (Fig. 7B) and immunofluorescence (Fig. 7C). In line with previously published studies^30–32^, we found that proIGF-1Eb and proIGF-1Ec partially accumulated in the nucleus of HEK293-transfected cells, while proIGF-1Ea was mainly localised into the ER (Fig. 7B and C). Notably, proIGF-1Eb and proIGF-1Ec did not appear to be accumulated in the cytosol, and the molecular weight of proIGF-1Eb or proIGF-1Ec in the ER and nuclear fractions corresponded to that of proIGF-1s without signal peptide (5.3 kDa) (Fig. 7B). These data suggest that proIGF-1Eb and proIGF-1Ec translocate to the nucleus from the ER and not from the cytoplasm. Accordingly, treatment with the proteasome inhibitor MG132 increased the nuclear accumulation of proIGF-1Eb and proIGF-1Ec, while their cytoplasmic level remained undetectable (Fig. 7D). Several substrates of the nuclear ubiquitin-proteasome system have been identified to date, showing that the nuclear ubiquitin-proteasome system is a key quality-control mechanism that rapidly eliminates unfolded or damaged proteins^33^. In this regard, the nuclear degradation of proIGF-1Eb and proIGF-1Ec might represent a mechanism for controlling the intracellular level of these highly disordered proteins; however, further studies are needed to clarify this point.

## Discussion

The mechanism by which IGF-1 production is regulated remains largely unknown^4^. In this study, we show the functional role of disordered E-peptides in the regulation of proIGF-1s production.

It is now well established that most peptide hormones and growth factors are initially synthesised as prohormones that are converted to active forms by endoproteolysis at specific sites^34^. Accordingly, the growth factor IGF-1 is produced as prohormone which contains a C-terminal domain, i.e. the E-domain, cleaved by furin convertase^3^. Evidence has been provided that only a small portion of proIGF-1 cleavage occurs intracellularly; hence most proIGF-1 might be converted to mature form at the cell surface membrane or extracellularly^6,7^. The results of our study confirm these findings showing that intracellular IGF-1 is mainly expressed as proIGF-1, not mature IGF-1, both *in vitro* and *in vivo* (Figs 2 and S2).

The amino acid composition of mature IGF-1 markedly differs compared to E-domains^5,35^. In particular, the E-domains were enriched in disorder-promoting amino acids, and by bioinformatic analyses, we demonstrated that the E-domains are IDRs, which were also confirmed by the limited proteolysis approach (Figs 1 and S1C). Therefore, the proIGF-1s consist of two different parts: the mature IGF-1, with a well-organised structure, and the flexible C-terminal E-domains. Notably, we verified that the disorder features of E-domains are conserved both across species and for all the three E-domains generated by alternative splicing^5^.

Many studies show that the conformational plasticity associated with intrinsic disorder provides IDRs with a complementary functional repertoire of ordered domains^15,17^, especially if these IDRs are at the protein termini^36^. Therefore, we next focused on the potential functional role of C-terminal Ea-, Eb- and Ec-domains.

The Ea-domain contains a highly conserved N-glycosylation site (N92) (Fig. 3A), which is heavily glycosylated with sugars comprising over 30% of the total mass of the proIGF-1Ea (Fig. 3B; see also^6,7^). Here, we demonstrated that the inhibition of N-glycosylation by site-directed mutagenesis (Fig. 3B), Tun treatment (Fig. 4A) or glucose withdrawal (Supplementary Fig. S5A), blocked the intracellular production of glycosylated proIGF-1Ea. The secretion of glycosylated proIGF-1Ea and mature IGF-1 was also substantially affected by the inhibition of N-glycosylation (Figs 3C, 4B and S5B). Accordingly, the conditioned media from IGF-1Ea-transfected cells treated with Tun were unable to activate the IGF-1R pathway in human MCF-7 breast cancer cell line (Fig. 4C). It is important to highlight that the unglycosylated proIGF-1Ea was not accumulated after N-glycosylation inhibition. In this line, we demonstrated that unglycosylated proIGF-1Ea had a faster turnover rate compared to glycosylated proIGF-1Ea (Fig. 5A). The proteasome inhibitor MG132 partially rescued the accumulation defect of unglycosylated proIGF-1Ea, but also markedly increased the production of a ~23 kDa band, probably representing unglycosylated proIGF-1Ea dimer (Fig. 5B,C and D). N-glycosylation influences the folding and trafficking of many glycoproteins ensuring protein solubility and minimising aggregation^37–39^. Accordingly, our work indicates that addition of N-glycan on Ea-domain prevents the degradation of proIGF-1Ea by the proteasome, probably overcoming the folding limitation of unglycosylated proIGF-1Ea and its entry into the ER-associated degradation (ERAD) pathway^40,41^. This finding has interesting functional implications since the IGF-1Ea isoform is widely expressed in normal tissues as well as in various tumour cells^5,8,10,42^. Thus, interfering with proIGF-1Ea glycosylation provides a means to regulate IGF-1 production. In this regard, we also investigated the effect of 2-DG on proIGF-1Ea glycosylation. 2-DG, a non-metabolizable glucose analogue, is one of the most frequently used antiglycolytic agents and it has come under increasing scrutiny as a therapeutic agent, especially in cancer^24^. However, since 2-DG also mimics mannose, might also interfere with N-linked glycosylation^24,26^. In fact, recent studies showed that interference with N-linked glycosylation rather than glycolysis as the predominant mechanism by which 2-DG inhibits cancer cell growth under normoxia^27–29^. Here, we demonstrated that 2-DG interferes with proIGF-1Ea glycosylation preventing the formation of the normal, highly glycosylated proIGF-1Ea (~17 kDa) (Fig. 6A). However, 2-DG also determined a concomitant dose-dependent increase of a ~12 kDa protein that probably represents an aberrant under-glycosylated proIGF-1Ea form. Notably, this protein was stable and efficiently secreted (Fig. 6B). Accordingly, despite the loss of highly glycosylated proIGF-1Ea after the 2-DG treatment, the condition media of these cells fully activated the IGF-1R of human breast cancer cell line MCF-7 (Fig. 6C). Thus, these data suggested that 2-DG interfere with normal proIGF-1Ea glycosylation process leading to an accumulation of an under-glycosylated form which is still able to fold and secreted by the cells. These results are in line with a recent study showing that the main effect of low, non-toxic and pharmacologically relevant concentration of 2-DG was to increase the incorporation of mannose on the glycan structure instead of inhibiting glycosylation^26^. Thus, 2-DG alters the glycosylation process favouring the synthesis of glycoproteins with incomplete or defective oligosaccharide chains. Taking advantage of the increased glucose uptake that occurs in most tumours, treatment with 2-DG might represent a new diagnostic and therapeutic tool to discriminate normal and cancer cells based on recognition of 2DG induced altered glycoproteins^26^. Further studies are needed to characterise the glycan structure of the 2-DG- induced under-glycosylated proIGF-1Ea and to test its potential role as a cancer biomarker.

We finally investigated if N-glycosylation has a functional role in proIGF-1Eb or proIGF-1Ec production. We should point out that in these prohormones two alternative E-domains, i.e. the Eb- and Ec-domains, replace the Ea-domain^3–5^. The IGF-1Eb and IGF-1Ec isoforms are not so widely expressed as IGF-1Ea, although their levels increased under specific conditions/stimuli (e.g. cancer^10,43^ or exercise^44^) and are also expressed in a species-specific manner suggesting isoform-specific functions^5^. Accordingly, in the present study, we demonstrated that the behaviour of proIGF-1Eb and proIGF-1Ec entirely differed compared to proIGF-1Ea. In particular, since both Eb- and Ec-domains lacked N-glycosylation sites, the production of proIGF-1Eb and proIGF-1Ec was unaffected by inhibition of N-glycosylation (Figs 7A and S8C). Moreover, the Eb- and Ec-domains determined the partial nuclear localisation of proIGF-1Eb and proIGF-1Ec (Fig. 7B and C; see also^30–32^). The mechanisms by which proIGF-1Eb and proIGF-1Ec enter into the nucleus after trafficking through the ER is still unknown^31^. As described for Ea-domain, the presence of the disordered Eb- and Ec- tails probably represents an obstacle for their correct folding and traffic through the ER. Accordingly, we previously demonstrated that the secretion of IGF-1 (both proIGF-1s and mature IGF-1) was significantly lower in the cell overexpressing IGF-1Eb or IGF-1Ec isoforms compared to IGF-1Ea-transfected cells^7^. Furthermore, the inhibition of proteasome by MG132 determined nuclear accumulation of proIGF-1Eb and proIGF-1Ec (Fig. 7D) demonstrating that the production of these prohormones was controlled through their subcellular localization.

In conclusion, our data suggested that alternative E-domains act as flexible tails controlling proIGF-1s and mature IGF-1 production. In particular, we demonstrated that N-linked glycosylation regulates the stability and secretion of proIGF-1Ea, probably ensuring proper prohormone folding and favouring its passage through the secretory pathway. Interference with proIGF-1Ea N-glycosylation (e.g. mutation of N-glycosylation site or Tun, glucose starvation and 2-DG treatment) directly affects protein IGF-1 level. The splice variants IGF-1Eb and IGF-1Ec encode the alternative proIGF-1Eb and proIGF-1Ec forms, which were insensitive to modulation of glycosylation. Notably, the Eb- and Ec- disordered tails promoted the nuclear accumulation of proIGF-1Eb and proIGF-1Ec, and thus directly affected the efficiency of proIGF-1s secretion.

Thus, disordered E-domains play a crucial role in the structure, regulation, localisation and functioning of IGF-1.

## Methods

### Tissue sampling and cell cultures

Human and animal tissues were previously obtained and analysed for mRNA extraction and IGF-1 isoforms quantification as described in Annibalini *et al*.^5^. From the same tissues stored at −80 °C, about 30 mg were used for protein extraction and subsequent western blotting analysis. Freshly frozen normal human adipose (2 males and 2 females, mean age 52 +/− 12 years) samples were provided by the complex structure of biomarkers (DOSMM) of National Cancer Institute of Milan. All subjects provided written informed consent before archival tissue samples. Pig (Sus scrofa; 3 males) and cow (Bos Taurus; 3 females) tissues were collected at local slaughter during routine meat inspection. The age of the animals ranged from 2 to 5 years. The study was conducted and reported in accordance with standards for reporting of diagnostic accuracy (STARD) requirements. The experimental protocols were in accordance with the Guide for the Care and Use of Laboratory Animals by Ministero della Sanità D.L. 116 (1992) and approved by the University of Urbino “Carlo Bo” Committee. The HEK293 and MCF-7 cell lines were obtained from the American Type Culture Collection (ATCC, Rockville, MD, USA). The cells lines were cultured in DMEM media supplemented with 10% fetal bovine serum, 2 mM L-glutamine, 1x MEM Non-essential Amino Acid Solution, 0.1 mg/ml streptomycin and 0.1 U/L penicillin. Cells were maintained in a humidified incubator (5% CO_2_) at 37 °C. All cell culture materials were purchased from Sigma-Aldrich (St. Louis, MO, USA).

### Protein extraction and western blotting analysis

Tissues and cells were processed for western blot analysis as previously reported^7^. Briefly, protein extracts were prepared by homogenizing with a Polytron homogenizer (KINEMATICA AG, Switzerland) in lysis buffer containing: 20 mM HEPES (pH 7.9), 25% v/v glycerol, 0.42 M NaCl, 0.2 mM EDTA, 1.5 mM MgCl2, 0.5% v/v Nonidet P-40, 1 mM DTT, 1 mM Naf, 1 mM Na_3_VO_4_, and 1 × complete protease inhibitor cocktail (Roche Diagnostics Ltd, Mannheim, Germany). The lysates were frozen and thawed twice and clarified by centrifugation at 12000 rpm for 10 minutes at 4 °C. Protein concentration in each sample was determined using the Bradford colourimetric assay. An equal amount of total proteins were fractionated by SDS-PAGE on a 15% polyacrylamide gel and then transferred to PVDF or nitrocellulose membranes (Bio-Rad Laboratories Inc). The membranes were incubated overnight at 4 °C with the primary antibodies directed towards: IGF-1 (1:2000; no. 500P11), purchased from Peprotech (Rocky Hill, New Jersey, USA), proIGF-1s (1:2000; no. PA5-19382) purchased from Invitrogen (Carlsbad, California, USA), β-tubulin (1:2000; no. 2146), lamin A/C (1:2000; no. 4777), calnexin (1:2000; no. 2679) phospho-IGF-1 Receptor β (1:2000; no. 3024), IGF-1 Receptor β (1:2000; no. 3027), phospho-p44/42 (ERK1/2) (1:2000; no. 9101), p44/42 (ERK1/2) (1:2000; no. 9102), phospho-Akt (Ser473) (1:2000; no. 9271) and Akt (1:2000; no. 9272) were purchased from Cell Signaling Technology (Beverly, MA, USA). Membranes were washed and incubated with appropriate secondary HRP-conjugated antibodies (Bio-Rad Laboratories Inc) at room temperature for 1 hour. After TBS-T washing, protein bands were visualised using Clarity Western ECL Substrate (Bio-Rad Laboratories Inc) and were quantified using Fluor-S MAX System (Bio-Rad Laboratories Inc) equipped with Quantity One software. β-tubulin was used for normalisation.

### Cell culture transfection assays

Transient cell transfection was carried out with TransIT-X2^®^ Transfection Reagent (Mirus Bio, Madison, WI, USA) with plasmid constructs containing sequences encoding proIGF-1s as previously described^7^. Each plasmid contained DNA encoding the class 1 IGF-1 48-amino acid signal peptide, the mature 70-amino acid IGF-1 peptide, the first 16 aa common in all E-domains, and C-terminal sequences encoding either the Ea (19 aa), the Eb (61 aa) or the Ec (24 aa) domain. Where indicated, to interfere with N-glycosylation, cells were grown in glucose-depleted medium or treated with 0.1 µg/ml of Tunicamycin (Tun) (Sigma-Aldrich, St. Louis, MO, USA) or 2-Deoxyglucose (2-DG) (Sigma-Aldrich, St. Louis, MO, USA) for 24 h. The supernatants of HEK293-transfected cells treated with Tun or 2-DG were also used to treat the MCF-7 cells for 1 h to evaluate their effects on IGF-1R, AKT and ERK1/2 phosphorylation. Afterwards, the MCF-7 cells were washed with PBS and lysed for western blotting analysis. Where indicated, the protein synthesis inhibitor cycloheximide (CHX) (Sigma-Aldrich) and the proteasome inhibitor MG132 (Sigma-Aldrich) were added to HEK293 cells cultured with a final concentration of 25 μg/ml and 10 μM respectively in a time-course experiment. The cells were then collected at indicated time points, lysed and prepared to western blotting analysis.

The efficiency of transfection was estimated by GFP-dependent fluorescence and by real-time RT-PCR for total IGF-1 mRNA levels at 24 hours after transfection, as previously described^7^.

Deglycosylation of proIGF-1Ea was performed by incubation of proIGF-1Ea enriched media with 2500 U of PNGase F (NewEngland Biolabs) for 3 hours at 37 °C, according to manufacturer’s recommendations. Aliquot of proIGF-1Ea supernatant incubated with equal volume of PNGase assay reaction buffer without the enzyme PNGase F was used as a control.

### Limited proteolysis

Supernatants of IGF-1Ea-, IGF-1Eb- or IGF-1Ec-transfected HEK293 cells were concentrated using an Amicon Ultra 3 K centrifugal filter unit (Merck Millipore, Billerica, MA, USA) and subjected to limited proteolysis. Limited proteolysis was performed by enzymatic digestion at 37 °C adding bovine trypsin (Sigma-Aldrich, Italy) or proteinase K (Sigma-Aldrich, Italy) to protein extract at a ratio 1:100 enzyme/substrate (w/w). Reactions were removed over a time-course, and the digested products were quenched with SDS sample buffer before SDS-PAGE and analysed by western blotting. The quantification of bands intensity was normalised to the “no trypsin” samples of each set.

### Conservation of Ea-domain N-glycosylation site

BLASTP was used to align protein sequence of human Ea-domain (EVHLKNASRGSAGNKNYRM) against the entire UniProt database using E-threshold of 0.01^45^. The obtained UniProt hits (250 sequences with a minimal sequence identity of ∼60%) were aligned using MUSCLE^46^, and WebLogo (http://weblogo.berkeley.edu/logo.cgi) was used to create relative frequency plots.

### Site-direct mutagenesis

Site-direct mutagenesis was used to generate the proIGF-1Ea^N92D^ mutant lacking the glycosylation site. The composition of PCR mutagenesis reactions run on an Applied Biosystems SimpliAmp Thermal Cycler were as follows: 25 μl of 2x Platinum SuperFi PCR Master Mix (Thermo Fisher); 10 μM of each primer (N92D-F 5′-ATTTGAAGGACGCAAGTAGAGGGAG-3′; 5′-N92D-R TTGCGTCCTTCAAATGTACTTCCTTC-3′) 1 ng plasmid contained DNA encoding proIGF-1Ea and H_2_O to 50 μl. Following PCR, the reactions were incubated with 1 μl (20 U) of Anza^TM^ 10 DpnI (Invitrogen) for 1 h to selectively digest the methylated parent plasmids, and the resulting PCR products were purified with GenElute PCR Clean-Up Kit (Sigma-Aldrich). 2 μl of the purified PCR reactions were transformed into electrocompetent E. coli XL1-Blue cells with selection for resistance to ampicillin (100 μg/ml), and successful mutagenesis was confirmed by sequencing of plasmid DNA.

### Subcellular localisation analysis

The cytosol, ER and nucleus isolations were performed as described in^47^. Briefly, cells were treated with permeabilisation buffer for 5 minutes and then were centrifuged at 3000 g for 5 min to collect the cytosol fraction in the soluble fraction. The pellet was then washed, subjected to lysis buffer for 5 minutes and centrifuged again at 3000 g for 5 min to collect the ER in the soluble fraction, whereas the pellet represented the nucleus fraction. Finally, all the samples were clarified at 7500 g for 10 minutes to remove cell debris and transferred to clean tubes for further analysis.

The HEK293 cells subjected to immunofluorescence were seeded in 4-well chamber slide at a density of 5 × 10^4^ cells/well, incubated overnight and transfected for IGF-1 isoforms expression as previously described. After overnight incubation, cells were fixed with 4% paraformaldehyde for 15 minutes, permeabilised with 0.2% TritonX-100, blocked with 5% of goat serum and incubated overnight at 4 °C with the anti-mature IGF-1 antibody (Prepotech). Next, cells were incubated 1 hour with an anti-rabbit-PE conjugated antibody, stained with DAPI, mounted with Fluoreshield (Sigma) and photographed with a fluorescence microscope (ZEISS AxioVert A.1).

### Statistical analysis

Data are represented as mean ± SEM of at least three independent experiments. Statistical analyses were performed using repeated measures ANOVA or one-way ANOVA as appropriate, followed by Bonferroni’s multiple comparison post hoc tests. A *p*-value < 0.05 was considered statistically significant.

### Data availability

The authors declare that the data supporting the findings of this study are available within the paper and its Supplementary Information files or upon reasonable request.

## Electronic supplementary material

Supplementary Information
