# Supplementary material for: The intrinsically disordered E-domains regulate the IGF-1 prohormones stability, subcellular localisation and secretion
Source: Sci Rep. 2018 Jun 11;8:8919. doi: 10.1038/s41598-018-27233-3 (PMC5995926; doi:10.1038/s41598-018-27233-3)
Supplement: Supplementary file 1 — Supplementary Information [file 41598_2018_27233_MOESM1_ESM.pdf]

Supplementary Information

The intrinsically disordered E-domains regulate the IGF-1 prohormones stability, subcellular localisation and secretion

Giosuè Annibalini[1]*, Serena Contarelli[1], Mauro De Santi[1], Roberta Saltarelli[1], Laura Di Patria[1], Michele Guescini[1], Anna Villarini[2], Giorgio Brandi[1], Vilberto Stocchi[1] and Elena Barbieri[1,3]

[1]Department of Biomolecular Sciences, University of Urbino Carlo Bo, 61029 Urbino, Italy.
[2]Research Department, Fondazione IRCCS Istituto Nazionale dei Tumori, 20133 Milan, Italy.
[3]IIM, Interuniversity Institute of Myology, 61029 Urbino, Italy.

* To whom correspondence should be addressed. Tel:+39 0722-303402 Fax:+39 0722-303401; Email: giosue.annibalini@uniurb.it

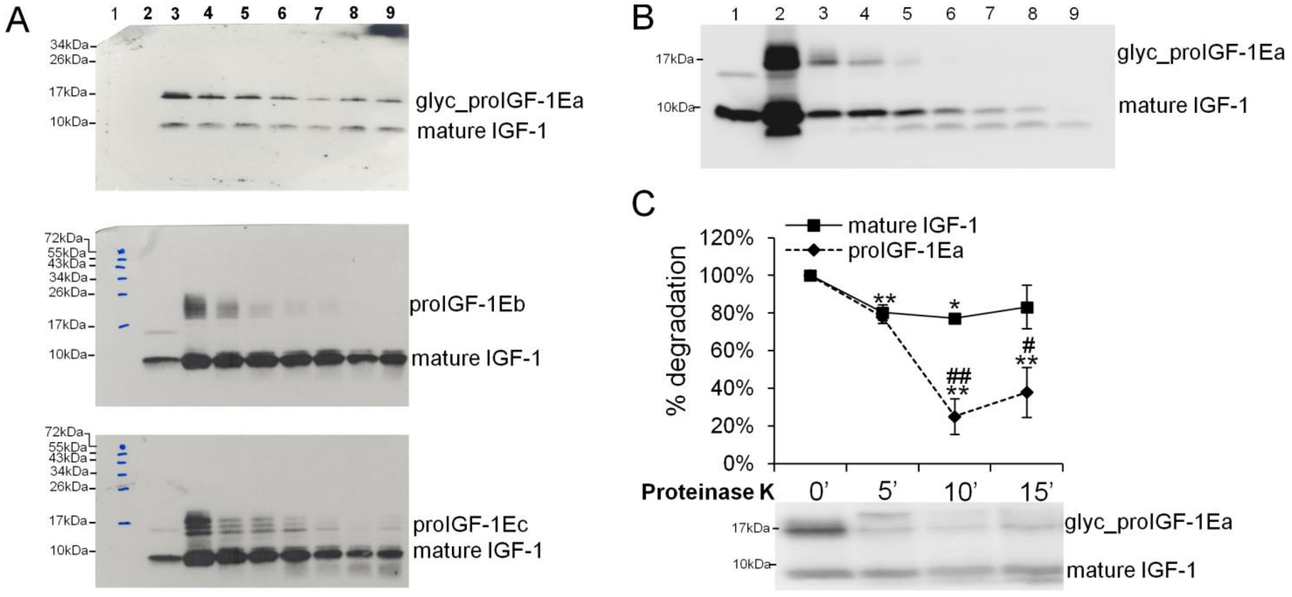

Supplementary Figure S1. Limited proteolysis of proIGF-1s with trypsin, original uncropped blots (A). Limited proteolysis of mature and glycosylated proIGF-1Ea with long-term trypsin incubation (B), or proteinase K digestion (C).

Cell culture supernatants of IGF-1Ea-, IGF-1Eb- or IGF-1Ec-transfected HEK293 cells were concentrated using an Amicon Ultra 3K centrifugal filter and incubated with trypsin (A and B) or proteinase K (C) at 37°C. Reactions were removed over a time-course, and the digested products were loaded on 12% SDS-PAGE and analysed by western blotting with an anti-mature IGF-1 antibody. Sample names are as follows: Figure S1A: 1: PageRuler Prestained Protein Ladder; 2: 25 ng of recombinant mature IGF-1 (Sigma-Aldrich I3769): 3-9: supernatants of IGF-1Ea-, IGF-1Eb- or IGF-1Ec-transfected HEK293 cells incubated with trypsin at 37°C for 0, 5, 10, 15, 20, 25 and 30 minutes. Figure S1B: 1: 25 ng of recombinant mature IGF-1 (Sigma-Aldrich I3769): 2: supernatants of IGF-1Ea transfected HEK293 cells (no trypsin); 3-9 supernatants of IGF-1Ea transfected HEK293 cells incubated with trypsin at 37°C for 0, 15, 30, 45, 60, 90 and 120 minutes. Figure S1C: Results are means ± SEM (n = 3). Repeated measures ANOVA, # ($p<0.01$) and ## ($p<0.0001$) significantly different compared to mature IGF-1; *($p<0.05$) and **($p<0.001$) significantly different compared to 0-minute time point. Samples that were included in cropped blots are indicated with bold numbers.

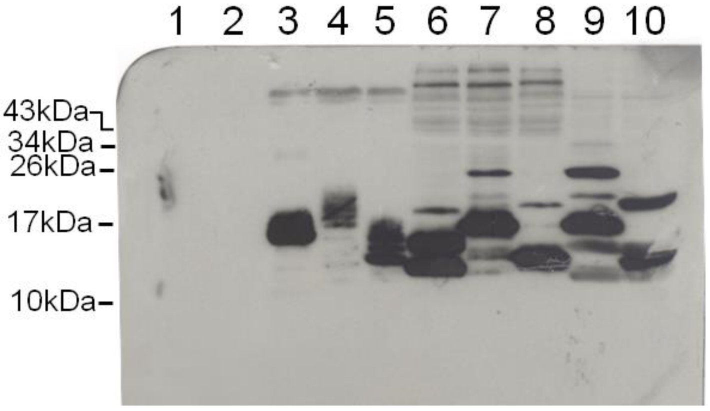

Supplementary Figure S2: Immunoblotting using an antibody directed against the common E-domain region of proIGF-1s (RSVRAQRHTD) (Invitrogen no. PA5-19382).

HEK293 cells were transfected with specific constructs and supernatants (lanes 3-5), and cell lysates (lanes 6-10) were analysed after 24 hours by western blot. Sample names are as follows: 1: PageRuler Prestained Protein Ladder; 2: 25 ng of recombinant mature IGF-1 (Sigma-Aldrich I3769); 3: cell culture supernatant from IGF-1Ea-transfected HEK293 cells; 4: cell culture supernatant from IGF-1Eb-transfected HEK293 cells; 5: cell culture supernatant from IGF-1Ec-transfected HEK293; 6: cell lysate from IGF-1Ea-transfected HEK293 cells; 7 and 9: cell lysate from IGF-1Eb-transfected HEK293 cells (technical replicates); 8 and 10: cell lysate from IGF-1Ec-transfected HEK293 cells (technical replicates).

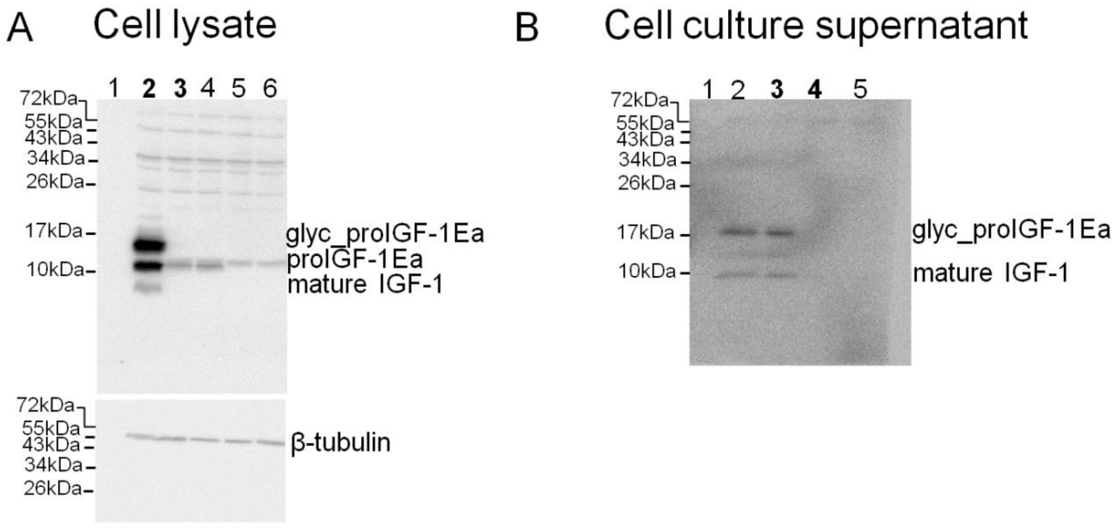

Supplementary Figure S3. Effect of Ea-domain N-glycosylation site mutation (IGF-1Ea^{N92D} mutant) on intracellular (A) or extracellular (B) proIGF-1Ea production. Original uncropped blots.

IGF-1Ea^{WT} and IGF-1Ea^{N92D} were transiently expressed in HEK293 cells. After 24 h the cell lysates (A) and cell culture supernatants (B) were analysed by western blot using an antibody directed against mature IGF-1 sequence. Sample names are as follows: Figure S3A; 1: PageRuler Prestained Protein Ladder; 2: cell lysate from IGF-1Ea^{WT}-transfected HEK293 cells; 3-6: cell lysate from IGF-1Ea^{N92D}-transfected HEK293 (biological replicates). Figure S3B; 1:PageRuler Prestained Protein Ladder; 2-3: cell culture supernatant from IGF-1Ea^{WT}-transfected HEK293 cells (biological replicates); 4-5: cell culture supernatant from IGF-1Ea^{N92D}-transfected HEK293 (biological replicates). Samples that were included in cropped blots are indicated with bold numbers.

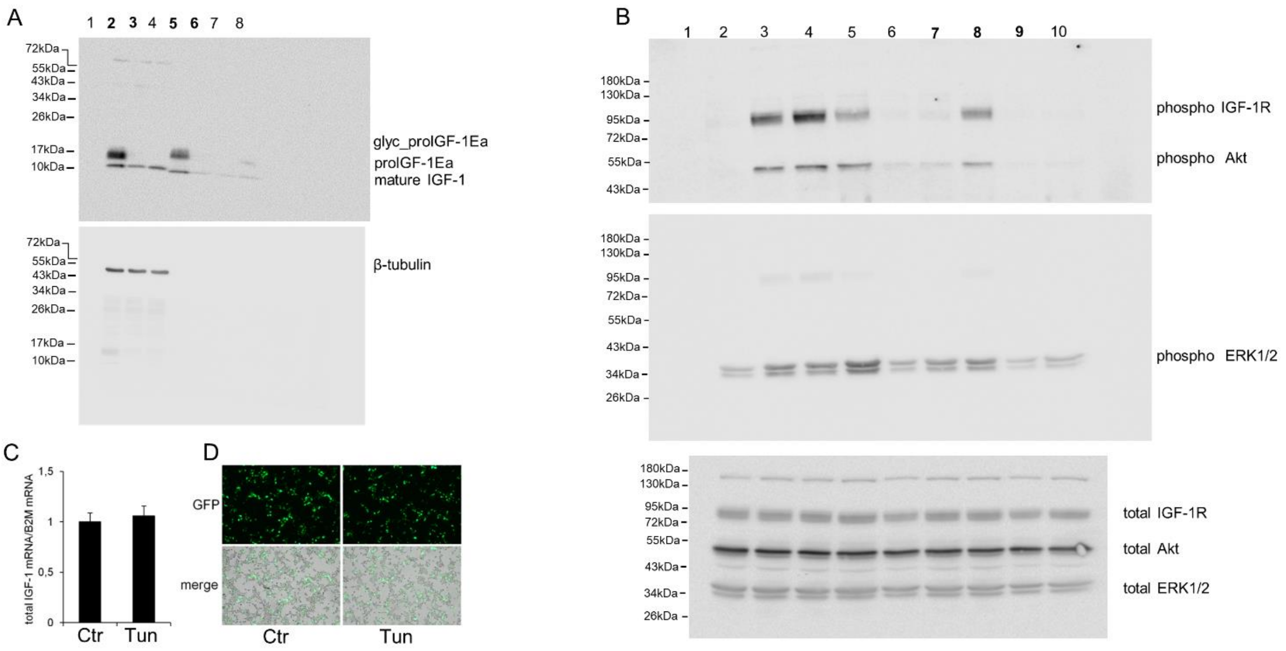

Supplementary Figure S4. Effects of tunicamycin (Tun) on proIGF-1Ea glycosylation, original uncropped blots (A and B). Total IGF-1 mRNA quantification (C) and GFP fluorescence (D) in IGF-1Ea-transfected HEK293 cells treated with Tun.

(A) IGF-1Ea was transiently expressed in HEK293 cells in the presence of 0.1 µg/ml of Tun. After 24 h the cell lysates (lanes 2-4) and cell culture supernatants (lanes 5-8) were analysed by western blot using an antibody directed against mature IGF-1 sequence. Sample names of Figure S4A are as follows: 1: PageRuler Prestained Protein Ladder; 2: cell lysate from untreated IGF-1Ea-transfected HEK293 cells; 3-4: cell lysate from IGF-1Ea-transfected HEK293 treated with 0.1 µg/ml of Tun (biological replicates); 5: cell culture supernatant from untreated IGF-1Ea-transfected HEK293 cells; 6-8: cell culture supernatant from IGF-1Ea-transfected HEK293 treated with 0.1 µg/ml of Tun (biological replicates). (B) Phosphorylation of IGF-1R, AKT and ERK1/2 after treatment of MCF-7 cells with cell culture supernatants from IGF-1Ea-transfected HEK293 cells treated with Tun. Sample names of Figure S4B are as follows: 1: PageRuler Prestained Protein Ladder; 2 and 7: treatment of MCF-7 cells with cell culture supernatants from empty vector-transfected HEK293 cells (biological replicates); 3-4: treatment of MCF7 cells with 25 ng of recombinant mature IGF-1 (biological replicates); 5 and 8: treatment of MCF-7 cells with cell culture supernatants from IGF-1Ea-transfected HEK293 cells (biological replicates); 6, 9 and 10: treatment of MCF-7 cells with cell culture supernatants from IGF-1Ea-transfected HEK293 treated with 0.1 µg/ml of Tun (biological replicates). (C and D) Comparison of transfection efficiency between IGF-1Ea-transfected HEK293 cells treated and untreated with Tun. No significant difference in total IGF-1 mRNA quantity (p=0.634) (C) or GFP fluorescence intensity (10x magnification) (D) was found. Samples that were included in cropped blots are indicated with bold numbers.

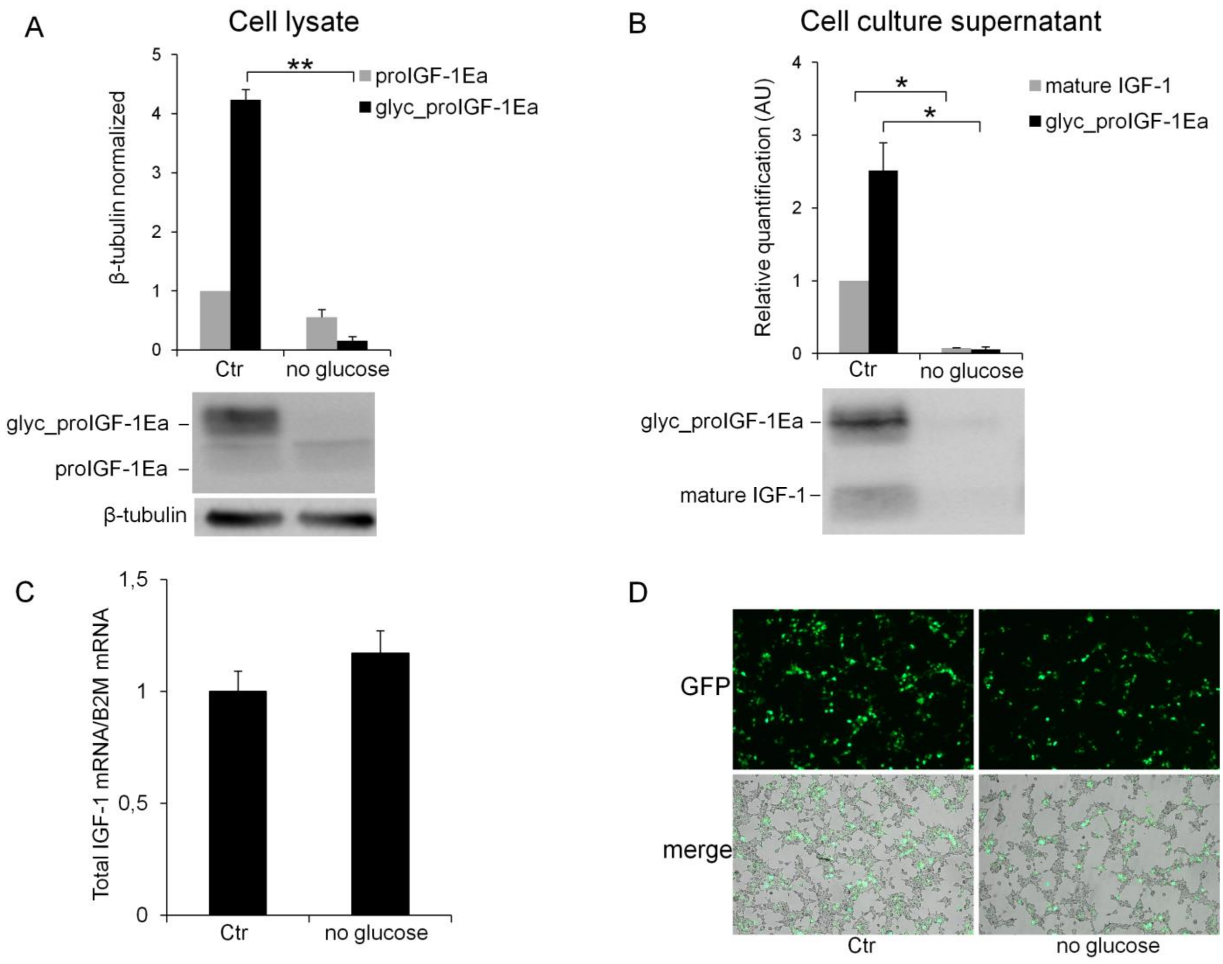

Supplementary Figure S5. Effects of glucose withdrawal on proIGF-1Ea glycosylation.

IGF-1Ea was transiently expressed in HEK293 cells in glucose-depleted medium (no glucose). After 24 h the cell lysates (A) and cell culture supernatants (B) were analysed by western blot and relative expression level of glycosylated proIGF-1Ea, unglycosylated proIGF-1Ea and mature IGF-1 was calculated. The band at a molecular weight around 17 kDa, corresponding to glycosylated proIGF-1A, disappeared in the absence of glucose in cell lysates (A) and the culture supernatants (B). The band corresponding to mature IGF-1 (~7kDa) was markedly reduced in the culture supernatants after glucose withdrawal (B). Results are means ± SEM (n= 3); T-test was used to evaluate statistical significance (*$p < 0.01$, **$p<0.0001$). β-tubulin was used as a loading control for the cell lysates. (C) Total IGF-1 mRNA quantification and (D) GFP fluorescence (10x magnification) in IGF-1Ea-transfected HEK293 cells grown in glucose-depleted medium. The mRNA expression of total IGF-1 (C) was unaffected by glucose withdrawal while glucose-depleted medium caused a slight decrease in cell number and GFP fluorescence (D).

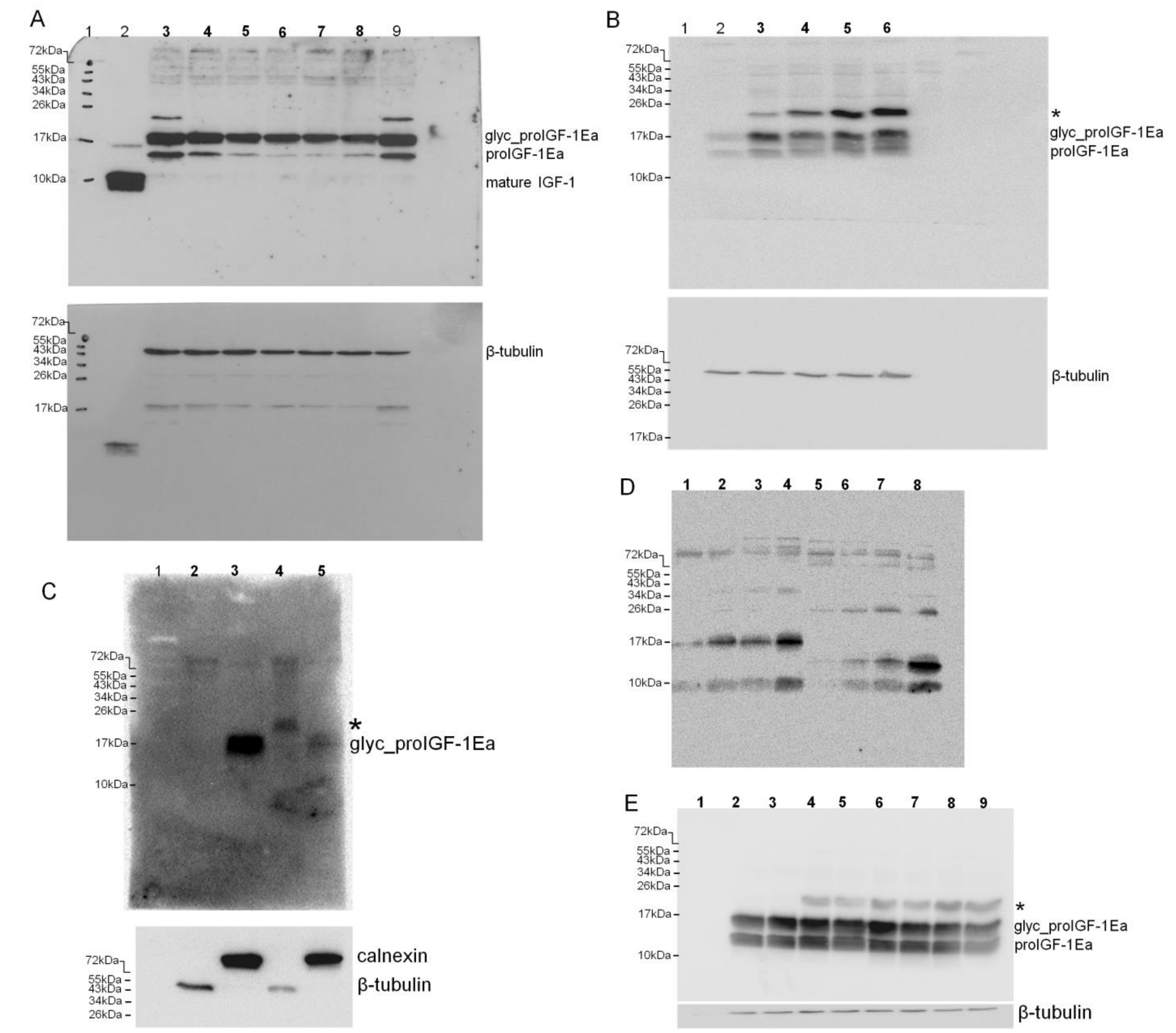

Supplementary Figure S6. Analysis of unglycosylated and glycosylated proIGF-1Ea turnover, original uncropped blots (A, B, C and D). Immunoblotting of IGF-1Ea-transfected HEK293 treated with MG132 using an antibody directed against the common E-domain region of proIGF-1s (E).

(A and B) IGF-1Ea was transiently expressed in HEK293 cells in the presence of 25μg/ml of protein synthesis inhibitor cycloheximide (CHX) (A) or 10μM of the proteasome inhibitor MG132 (B) in a time-course experiment. Cytosol (Cyt) and endoplasmic reticulum (ER) isolations of IGF-1Ea-transfected HEK293 cells treated with MG132 were shown in figure S6C. Deglycosylation of proIGF-1Ea enriched media using the *N*-Glycosidase F (PNGase F) was displayed in figure S6D. Immunoblotting of IGF-1Ea-transfected HEK293 treated with 10μM MG132 using an antibody directed against common region of E-peptides (E).Sample names of Figure S6A are as follows: 1: PageRuler Prestained Protein Ladder; 2: 25 ng of recombinant mature IGF-1 (Sigma-Aldrich I3769); 3-7: cell lysate from IGF-1Ea-transfected HEK293 treated with 25μg/ml of CHX for 0’, 30’, 60’, 90’ and 120’ respectively; 8: cell lysate from IGF-1Ea-transfected HEK293 co-treated with CHX and MG132 (10μM) for four hours; 9: technical replicate of sample 3. Sample names of Figure S6B are as follows: 1: PageRuler Prestained Protein Ladder; 2: cell lysate from untreated IGF-1Ea-transfected HEK293 cells; 3-6: cell lysate from IGF-1Ea-transfected HEK293 treated with 10μM of MG132 for 0, 2, 4 and 6 hours. After MG132 treatment intracellular accumulation of a ~23kDa band was found (indicated with an asterisk in figure S6B), probably representing unglycosylated proIGF-1Ea dimer. Sample names of Figure S6C are as follows: 1: PageRuler Prestained Protein Ladder; 2: Cyt fraction of IGF-1Ea-transfected HEK293 cells; 3: ER fraction of IGF-1Ea-transfected HEK293 cells; 4: Cyt fraction of IGF-1Ea-transfected HEK293 cells treated with 10μM of MG132 for 6h; 5: ER of IGF-1Ea-transfected HEK293 cells treated with 10μM of MG132 for 6h. Sample names of Figure S6D are as follows: 1-4: 10, 20, 30 and 40 μg of cell culture supernatants of IGF-1Ea-transfected HEK293; 5-8: 10, 20, 30 and 40 μg of cell culture supernatants of IGF-1Ea-transfected HEK293 after PNGase deglycosylation. Sample names of Figure S6E are as follows: 1:

PageRuler Prestained Protein Ladder; 2-3: cell lysate from untreated IGF-1Ea-transfected HEK293 cells (technical replicates); 4-5: cell lysate from IGF-1Ea-transfected HEK293 treated with 10μM of MG132 for 2 hours (technical replicates); 6-7: cell lysate from IGF-1Ea-transfected HEK293 treated with 10μM of MG132 for 4 hours (technical replicates); 8-9: cell lysate from IGF-1Ea-transfected HEK293 treated with 10μM of MG132 for 6 hours (technical replicates). Samples that were included in cropped blots are indicated with bold numbers.

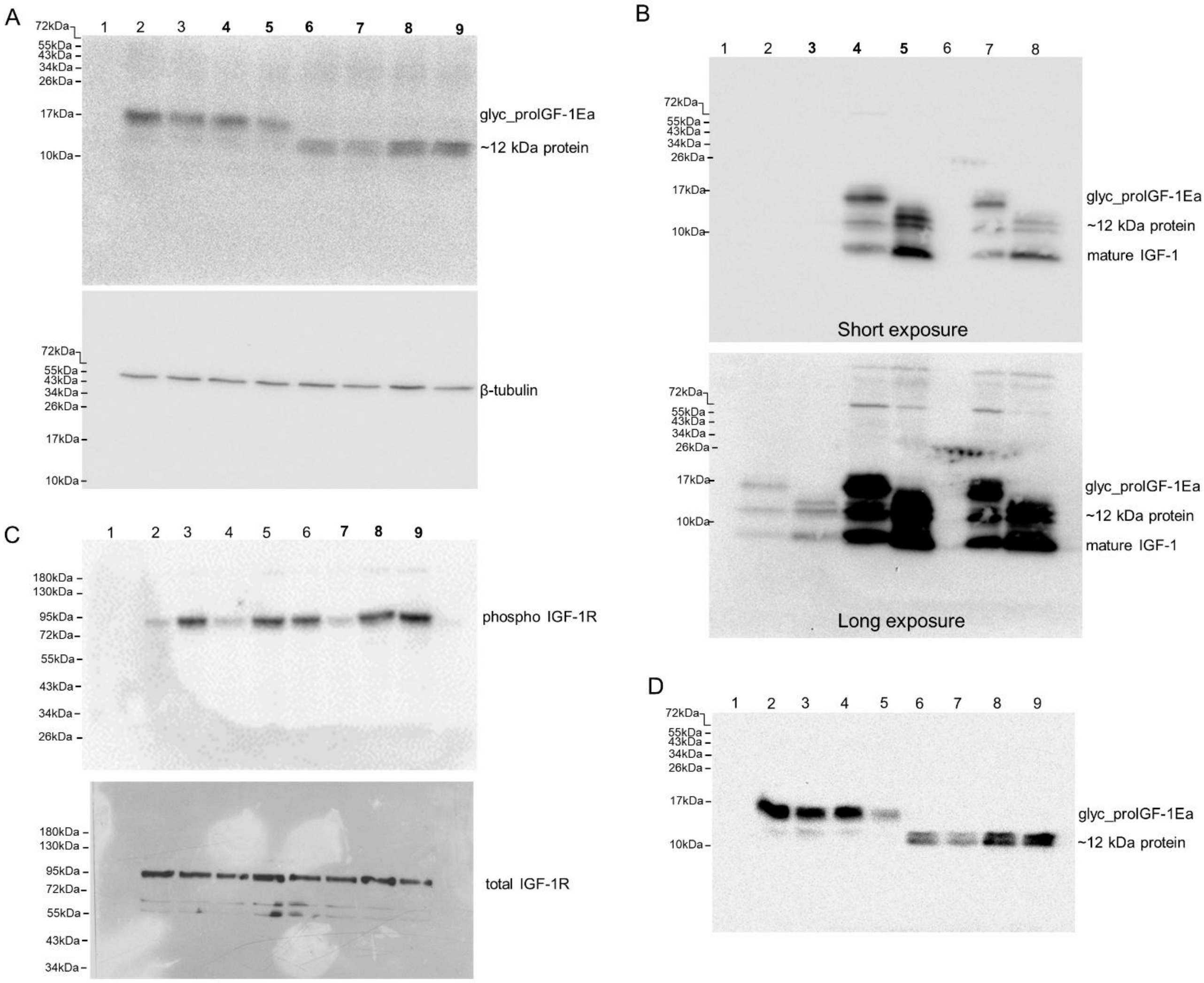

Supplementary Figure S7. Effect of 2-Deoxyglucose (2-DG) on glycosylated proIGF-1Ea production, original uncropped blots (A, B and C). Immunoblotting of IGF-1Ea-transfected HEK293 treated with 2-DG using an antibody directed against the common E-domain region of proIGF-1s (D).

IGF-1Ea was transiently expressed in HEK293 cells grown in low glucose medium (0.65 g/L) with or without 2-DG. After 24 h the cell lysates (A) and cell culture supernatants (B) were analysed by western blot using an antibody directed against mature IGF-1 sequence. Phosphorylation of IGF-1R after treatment of MCF-7 cells with cell culture supernatants from IGF-1Ea-transfected HEK293 cells treated or not with 0.2 g/L of 2-DG (C). Immunoblotting of IGF-1Ea-transfected HEK293 treated with 2-DG using an antibody directed against common region of E-peptides (D). Sample names of Figure S7A and S7D are as follows: 1: PageRuler Prestained Protein Ladder; 2-5: cell lysate from IGF-1Ea-transfected HEK293 (biological replicates); 6-7: cell lysate from IGF-1Ea-transfected HEK293 treated with 0.2 g/L of 2-DG (biological replicates); 8-9: cell lysate from IGF-1Ea-transfected HEK293 treated with 0.75 g/L of 2-DG (biological replicates). Sample names of Figure S7B are as follows: 1: PageRuler Prestained Protein Ladder; 2, 4 and 7: cell culture supernatants from IGF-1Ea-transfected HEK293 (biological replicates); 3, 5 and 8: cell culture supernatants from IGF-1Ea-transfected HEK293 treated with 0.2 g/L of 2-DG (biological replicates). Sample names of Figure S7C are as follows: 1: PageRuler Prestained Protein Ladder; 2, 4 and 7: treatment of MCF-7 cells with cell culture supernatants from empty vector-transfected HEK293 cells (biological replicates); 3: treatment of MCF-7 cells with 25 ng of recombinant mature IGF-1; 5 and 8: treatment of MCF-7 cells with cell culture supernatants from IGF-1Ea-transfected HEK293 cells (biological replicates); 6 and 9 treatment of MCF-7 cells with cell culture supernatants from IGF-1Ea-transfected HEK293 cells treated with 0.2 g/L of 2-DG (biological replicates). Samples that were included in cropped blots are indicated with bold numbers.

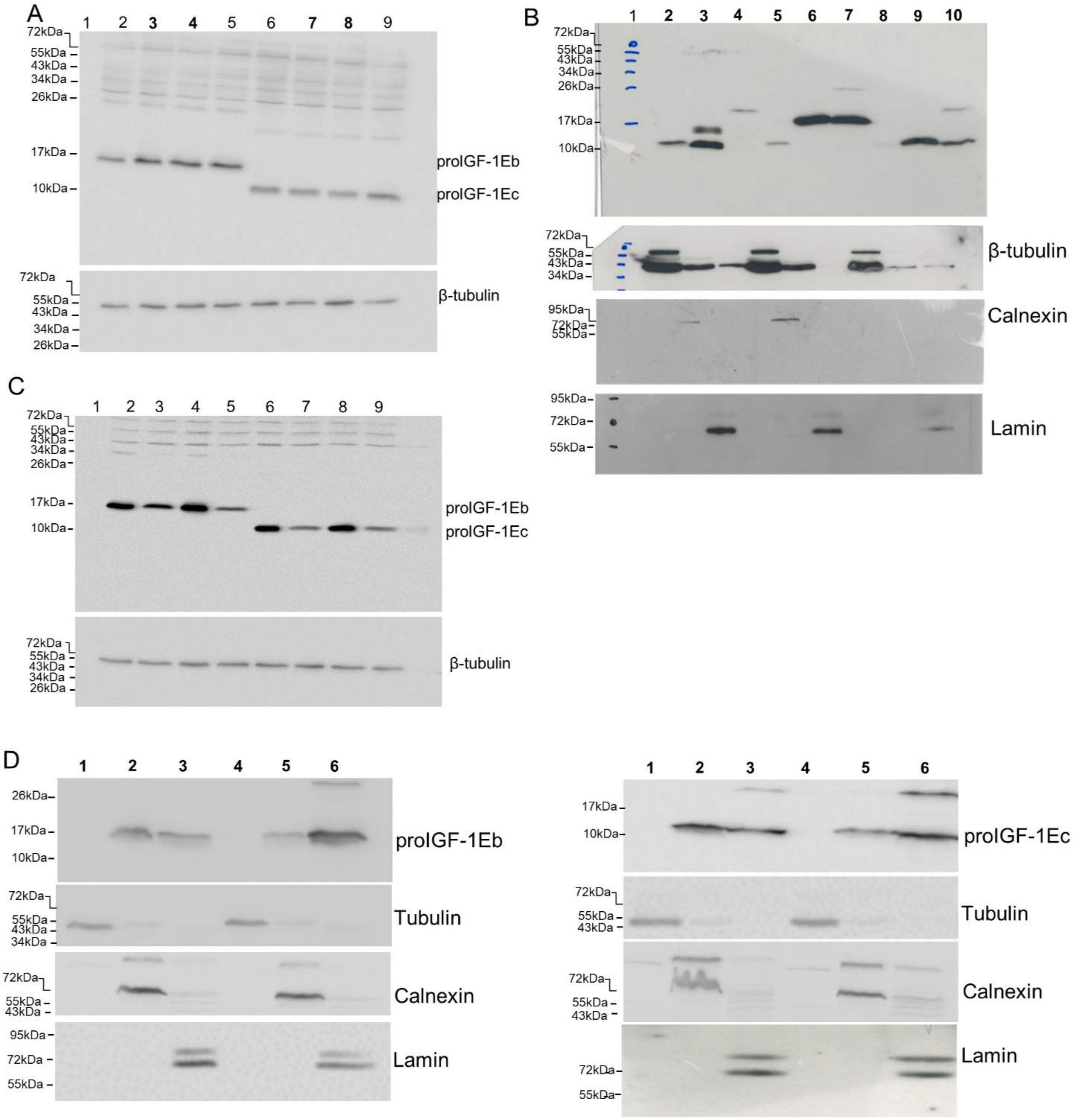

Supplementary Figure S8. Effect of N-glycosylation inhibitor tunicamycin (Tun) on IGF-1Eb and IGF-1Ec production (A) and subcellular localisation of proIGF-1s in untreated (B) or MG132 treated HEK293 cells (D), original uncropped blots. Effects of glucose withdrawal on proIGF-1Eb and proIGF-1Ec production (C).

(A) IGF-1Eb (lanes 2-5) or IGF-1Ec (lanes 6-9) was transiently expressed in HEK293 cells in the presence of 0.1 µg /ml of tunicamycin (Tun). After 24 h the cell lysates were analysed by western blot using an antibody directed against mature IGF-1 sequence. Sample names of Figure S8A are as follows: 1: PageRuler Prestained Protein Ladder; 2-3: cell lysate from IGF-1Eb-transfected HEK293 (biological replicates); 4-5: cell lysate from IGF-1Eb-transfected HEK293 treated with 0.1 µg/ml Tun (biological replicates); 6-7: cell lysate from IGF-1Ec-transfected HEK293 (biological replicates); 8-9: cell lysate from IGF-1Ec-transfected HEK293 treated with 0.1 µg/ml Tun (biological replicates). β-tubulin was used as a loading control for the cell lysates. (B) Subcellular localisation of IGF-1 isoforms analysed by cytosol (Cyt), endoplasmic reticulum (ER) and nucleus isolations. Sample names of Figure S8B are as follows: 1: PageRuler Prestained Protein Ladder; 2, 5 and 8: Cyt fraction of HEK293 cells transfected with IGF-1Ea, IGF-1Eb and IGF-1Ec isoforms respectively; 3, 6 and 9: ER fraction of HEK293 cells transfected with IGF-1Ea, IGF-1Eb and IGF-1Ec isoforms respectively; 4, 7 and 10: nuclear fraction of HEK293 cells transfected with IGF-1Ea, IGF-1Eb and IGF-1Ec isoforms respectively. β-tubulin was used as a loading control for the Cyt separation; calnexin as a control for

the ER separation and lamin as a control for the nucleus separation. Samples that were included in cropped blots are indicated with bold numbers. (C) IGF-1Eb (lanes 2-5) or IGF-1Ec (lanes 6-9) was transiently expressed in HEK293 cells grown in normal or glucose-depleted medium (no glucose). After 24 h the cell lysates were analysed by western blot using an antibody directed against mature IGF-1 sequence. β-tubulin was used as a loading control for the cell lysates. Sample names of Figure S8C are as follows: 1: PageRuler Prestained Protein Ladder; 2 and 4: cell lysate from IGF-1Eb-transfected HEK293 cells (biological replicates); 3-5: cell lysate from IGF-1Eb-transfected HEK293 cells grown in glucose-depleted medium (biological replicates); 6-8: cell lysate from IGF-1Ec-transfected HEK293 cells (biological replicates); 7-9: cell lysate from IGF-1Ec-transfected HEK293 cells grown in glucose-depleted medium (biological replicates). (D) Subcellular localisation of proIGF-1Eb (left panel) or proIGF-1Ec (right panel) after treatment with 10μM of the proteasome inhibitor MG132 for 6h. Sample names of Figure S8D are as follows: 1, 2 and 3: Cyt, ER and nuclear fraction of HEK293 cells transfected with IGF-1Eb (left panel) or IGF-1Ec (right panel); 4, 5 and 6 Cyt, ER and nuclear fraction of HEK293 cells transfected with IGF-1Eb (left panel) or IGF-1Ec (right panel) and treated with the proteasome inhibitor MG132. β-tubulin was used as a loading control for the Cyt separation; calnexin as a control for the ER separation and lamin as a control for the nucleus separation.
